# Biogeographical and Biodiversity Patterns of Marine Planktonic Bacteria Spanning from the South China Sea across the Gulf of Bengal to the Northern Arabian Sea

**DOI:** 10.1128/spectrum.00398-23

**Published:** 2023-04-26

**Authors:** Lijuan Ren, Xingyu Song, Chuangfeng Wu, Gang Li, Xiufeng Zhang, Xiaomin Xia, Chenhui Xiang, Bo-Ping Han, Erik Jeppesen, Qinglong L. Wu

**Affiliations:** a Department of Ecology and Institute of Hydrobiology, Jinan University, Guangzhou, China; b Key Laboratory of Tropical Marine Bio-resources and Ecology and Key Laboratory of Science and Technology on Operational Oceanography, South China Sea Institute of Oceanology, Chinese Academy of Sciences, Guangzhou, China; c Sino-Danish Centre for Education and Research, University of Chinese Academy of Sciences, Beijing, China; d Department of Bioscience, Aarhus University, Silkeborg, Denmark; e Limnology Laboratory, Department of Biological Sciences and Centre for Ecosystem Research and Implementation, Middle East Technical University, Ankara, Turkey; f Center for Evolution and Conservation Biology, Southern Marine Sciences and Engineering Guangdong Laboratory (Guangzhou), Guangzhou, China; g State Key Laboratory of Lake Science and Environment, Nanjing Institute of Geography and Limnology, Chinese Academy of Sciences, Nanjing, China; Institut Ruder Boskovic

**Keywords:** biogeography, marine planktonic bacteria, diversity pattern, niche breadth, species sorting

## Abstract

Understanding the biogeographical and biodiversity patterns of bacterial communities is essential in unraveling their responses to future environmental changes. However, the relationships between marine planktonic bacterial biodiversity and seawater chlorophyll *a* are largely understudied. Here, we used high-throughput sequencing to study the biodiversity patterns of marine planktonic bacteria across a broad chlorophyll *a* gradient spanning from the South China Sea across the Gulf of Bengal to the northern Arabian Sea. We found that the biogeographical patterns of marine planktonic bacteria complied with the scenario of homogeneous selection, with chlorophyll *a* concentration being the key environmental selecting variable of bacteria taxa. The relative abundance of *Prochlorococcus*, the SAR11 clade, the SAR116 clade, and the SAR86 clade significantly decreased in habitats with high chlorophyll *a* concentrations (>0.5 μg/L). Free-living bacteria (FLB) and particle-associated bacteria (PAB) displayed contrasting alpha diversity and chlorophyll *a* relationships with a positive linear correlation for FLB but a negative correlation for PAB. We further found that PAB had a narrower niche breadth of chlorophyll *a* than did FLB, with far fewer bacterial taxa being favored at higher chlorophyll *a* concentrations. Higher chlorophyll *a* concentrations were linked to the enhanced stochastic drift and reduced beta diversity of PAB but to the weakened homogeneous selection, enhanced dispersal limitation, and increased beta diversity of FLB. Taken together, our findings might broaden our knowledge about the biogeography of marine planktonic bacteria and advance the understanding of bacterial roles in predicting ecosystem functioning under future environmental changes that are derived from eutrophication.

**IMPORTANCE** One of the long-standing interests of biogeography is to explore diversity patterns and uncover their underlying mechanisms. Despite intensive studies on the responses of eukaryotic communities to chlorophyll *a* concentrations, we know little about how changes in seawater chlorophyll *a* concentrations affect free-living bacteria (FLB) and particle-associated bacteria (PAB) diversity patterns in natural systems. Our biogeography study demonstrated that marine FLB and PAB displayed contrasting diversity and chlorophyll *a* relationships and exhibited completely different assembly mechanisms. Our findings broaden our knowledge about the biogeographical and biodiversity patterns of marine planktonic bacteria in nature systems and suggest that PAB and FLB should be considered independently in predicting marine ecosystem functioning under future frequent eutrophication.

## INTRODUCTION

One of the long-standing interests of biogeography is to explore diversity patterns and uncover their underlying mechanisms (1, 2). The chlorophyll *a* concentration is considered to be an important factor in relation to the diversity patterns of eukaryotic plankton communities in marine ecosystems (3–6), and it is widely accepted that there is a positive linear or hump-shaped relationship between the seawater chlorophyll *a* concentration and diversity (7, 8). Such relationships are based on the ecological effects of “complementarity” and “sampling” (9, 10). The complementary effect assumes that more diverse communities support higher primary productivity, which are primarily indicated as the chlorophyll *a* concentration here, because of species niche differentiation and, thus, functional complementarity to utilize resources more completely. Meanwhile, the sampling effect suggests that species that are more productive and competitively dominant are more likely to be found in species-rich communities (11). However, primary productivity might also have no impact on diversity if, for instance, the productive habitat is dominated by several key species that are present at low diversity (12).

Compared with eukaryotic communities, the relationships between marine planktonic bacterial biodiversity and seawater chlorophyll *a* concentration are largely understudied, although bacterial communities act as good models with which to test ecological theory (7, 13, 14). Currently, only a few studies have explored the relationships between marine bacterial diversity and the seawater chlorophyll *a* concentration in natural communities (15–19). Some of the existing studies have shown that marine planktonic bacteria, despite their small size and efficient dispersal (20, 21), may tend to exhibit regular patterns, resembling those observed in eukaryotic plankton communities (16–18, 22–27). In marine ecosystems, bacteria are ubiquitous and abundant (15, 21). They typically form complex communities, and their diversity, interactions, structure, and functions are critical to ecosystem functioning and service (15, 21). Therefore, understanding patterns of bacterial communities is essential in unraveling the responses of marine ecosystems to future environmental changes.

There are two life styles of planktonic bacteria, namely, particle-attached bacteria (PAB) and free-living bacteria (FLB), and the two coexist in the ocean with obvious niche differentiation (28, 29). PAB are associated with a copiotrophic lifestyle, whereas FLB prefer an oligotrophic lifestyle and streamlined genomes (28, 30, 31). In the surface seawaters, the copiotrophic lifestyle PAB are mainly affiliated with *Bacteriodetes*, *Gammaproteobacteria*, *Planctomycetes*, and *Verrucomicrobia*, whereas the oligotrophic lifestyle FLB are dominated by SAR 11, SAR 86, SAR 406, and SAR 202 (25, 26, 31, 32). Therefore, to gain a deeper understanding of the diversity patterns of planktonic marine bacteria and the spatial-temporal dynamics of community structure in response to environmental changes, PAB and FLB should be studied separately. However, quite a few studies regarding the seawater chlorophyll *a* concentration and bacterial diversity relationships distinguish between PAB and FLB or neglect the fraction of PAB through the prefiltration of water particles (31–34). We know little about how or even if PAB and FLB diversity responds to changes in the seawater chlorophyll *a* concentration and the underlying ecological processes. The ecological processes underlying marine bacterial biogeographic patterns can be uncovered via the community assembly framework, which is an effective and robust tool that can be used to quantify bacterial community assembly processes, including homogeneous and heterogeneous selection, dispersal limitations, homogenizing dispersal, and stochastic drift fractions (35).

By performing the community assembly framework, a recent study spanning from the subantarctic to subarctic regions in the Pacific Ocean revealed that the ecological processes of homogeneous and heterogeneous selection, dispersal, and drift played different roles on the community assembly of PAB and FLB along the 12,400 km transect (25). Homogeneous selection was found to have predominant significance in the FLB community assembly, whereas dispersal limitation and drift displayed relatively higher significance, compared to selection, in the PAB community assembly (25). Different ecological processes are seen as complementary, rather than mutually exclusive, in the shaping of the biogeographic patterns of the bacterial communities (23, 25, 36). Supposing that the seawater chlorophyll *a* concentration and both PAB and FLB diversity have significant correlations (i.e., a strong effect of selection), the dispersal of individuals and drift might also contribute to the PAB and FLB community assembly patterns across a chlorophyll *a* gradient (23, 37–39).

**FIG 1:**
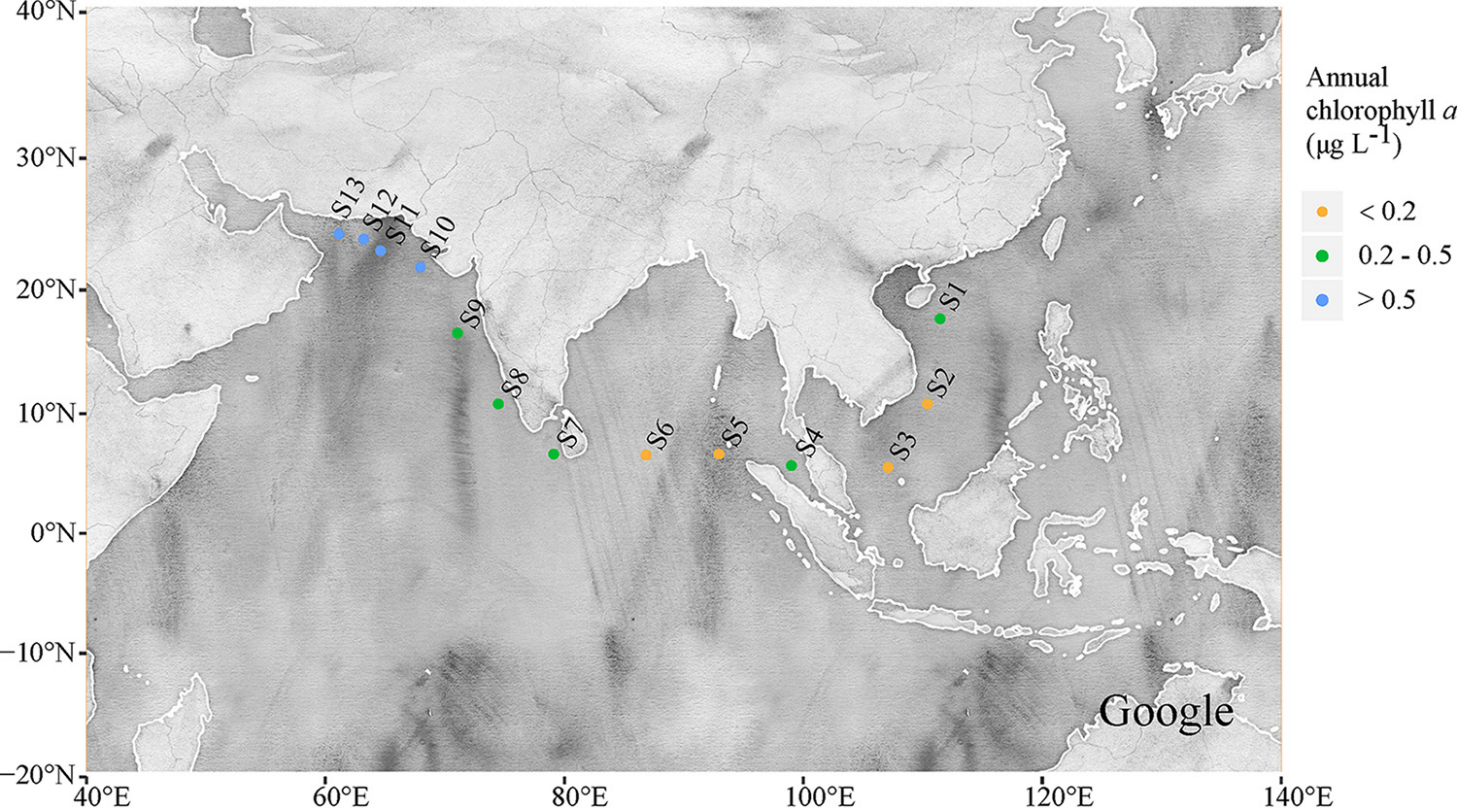
The locations of the sampling sites. The sites were colored by the annual chlorophyll *a* before the sampling time. The data were obtained from the NASA MODIS ocean color website (https://oceancolor.gsfc.nasa.gov/).

Here, we used the community assembly framework to study the biogeographical and biodiversity patterns of marine planktonic PAB and FLB across a broad chlorophyll *a* gradient (i.e., the annual chlorophyll *a* concentration from 0.12 to 2.89 μg/L), spanning from the South China Sea across the Gulf of Bengal to the northern Arabian Sea (Fig. 1). High chlorophyll *a* concentrations were found in the northern Arabian Sea (Fig. 1, sites 10 to 13). Our previous studies reported that there was a prevalence of Noctiluca scintillans (dinoflagellate), which replaced diatoms as the main bloom species during winter in the northern Arabian Sea (40, 41). Massive blooms of *Noctiluca scintillans* have been reported to frequently and widely occur in the Arabian Sea and in other parts of the Indian Ocean (41–44), and they seriously threaten the diversity of marine animals, phytoplankton, and zooplankton due to oxygen depletion, potential ammonium toxicity, and intraspecific and interspecific competition (44, 45). Therefore, we hypothesized that (i) massive blooms of *Noctiluca scintillans* might also affect the biogeographical and biodiversity patterns of PAB and FLB; (ii) marine planktonic bacterial (PAB and FLB) diversity exhibits similar relationships to those observed for eukaryotes, such as positive linear or hump-shaped correlations with the seawater chlorophyll *a* concentration; and (iii) the biogeographical and biodiversity patterns of both PAB and FLB across a broad chlorophyll *a* gradient will be dominantly shaped by the combination of selection, dispersal, and stochastic drift fractions.

## RESULTS

### Physicochemical and nutrient characteristics.

The measured chlorophyll *a* concentration, ranging from 0.13 to 5.31 μg/L across the sampling sites, was positively correlated with the annual chlorophyll *a* concentration, annual particulate organic carbon, salinity, silicate (SiO_3_^2−^), phosphorus (PO_4_^3−^), total nitrogen (TN), nitrite (NO_2_^−^), and nitrate (NO_3_^−^) (Spearman's rank correlation, *r* > 0.561; *P* < 0.05) (Table S1), but it was negatively linked to the measured seawater temperature, annual temperature, and ammonia (NH_4_^+^) (Spearman's rank correlation, *r* < −0.335; *P* < 0.05) (Table S1). A principal-components analysis (PCA) showed that the investigated environmental variables could be reduced to two principal components (PC1 and PC2) that described 69.7% and 10.7% of the total variation of the variables, respectively (Fig. S1). Most of the environmental variables were significantly related to each other (Table S1). Moreover, significant positive correlations of the measured chlorophyll *a* concentration with PO_4_^3−^, SiO_3_^2−^, NO_3_^−^, NO_2_^−^, and TN (Spearman's rank correlation, *r* > 0.561; *P* < 0.01) suggested strong relationships between the measured chlorophyll *a* concentration and nutrient concentrations (Table S1).

### Alpha diversity patterns of both PAB and FLB.

**FIG 2:**
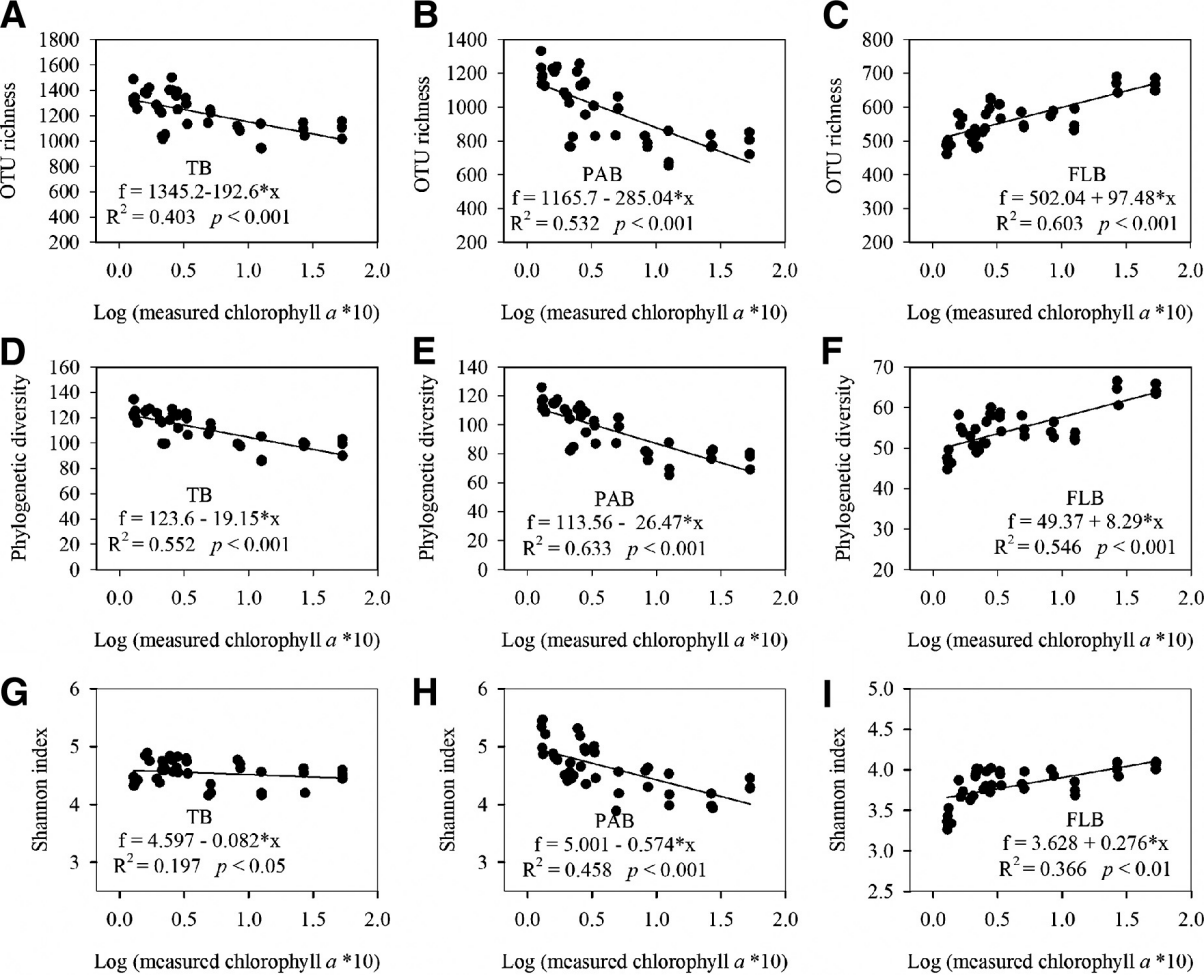
The alpha diversity of the OTU richness, Faith’s phylogenetic diversity (Faith’s PD), and Shannon index of the total bacteria (TB) (A, D, and G), particle-associated bacteria (PAB) (B, E, and H), and free-living bacteria (FLB) (C, F, and I) along the gradient of the measured chlorophyll *a* concentrations.

The planktonic bacterial diversity was assessed by using high-throughput sequencing of the V4 region of the 16S rRNA gene. From the entire sample set, we obtained 2,799,147 quality sequences, ranging from 20,554 to 42,300 per sample. After quality filtering and the removal of chimeric sequences, a total of 42 phyla (subphyla) were identified (Fig. S2A). Between PAB and FLB, significantly different compositions were observed (PERMANOVA: *F* = 52.14, *P* < 0.001). PAB was mainly composed of *Bacteroidetes* (primarily *Flavobacteriaceae*), *Cyanobacteria* (primarily *Synechococcus*), *Alphaproteobacteria* (primarily *Rhodobacteraceae*), *Gammaproteobacteria* (primarily *Moraxellaceae*), and *Planctomycetes*, whereas FLB was dominated by *Alphaproteobacteria* (primarily the SAR11 clade), *Cyanobacteria* (primarily *Synechococcus* and *Prochlorococcus*), *Actinobacteria* (primarily the OM1 clade), and *Bacteroidetes* (primarily *Flavobacteriaceae*) (Fig. S2A and B). In addition, significantly higher alpha diversity was observed for PAB than for FLB (*t* test: *t* > 10.76, *P* < 0.05) (Fig. S3A–F). When relating the investigated environmental variables to the alpha diversity patterns, we found that the measured chlorophyll *a* concentrations had high correlations with the alpha diversity (i.e., OTU richness, Faith’s phylogenetic diversity, and Shannon index) of both PAB and FLB (Table 1). We further found that the alpha diversity of both the total bacteria and PAB decreased linearly with an increasing seawater chlorophyll *a* concentration (R^2^ > 0.197, *P* < 0.05) (Fig. 2A, B, D, E, G, and H). In contrast, the FLB alpha diversity increased linearly with the seawater chlorophyll *a* concentration (R^2^ > 0.366, *P < *0.01) (Fig. 2C, F, and I).

**TABLE 1:** Generalized linear model analyses relating the investigated environmental variables to the alpha diversity (OTU richness, Faith’s PD and Shannon index) patterns of the total bacteria, particle-associated bacteria (PAB), and free-living bacteria (FLB)^*a*^

Index	OTU richness			Faith’s PD			Shannon index		
	Total bacteria	PAB	FLB	Total bacteria	PAB	FLB	Total bacteria	PAB	FLB
Chlorophyll *a* (μg/L)	−3.79**	−3.83**	6.76**	−2.79**	−4.37**	−2.79**	0.78	−3.17**	3.29*
PO_4_^3−^ (μmol L^−1^)	−3.52**	−1.59	−4.15**	−2.31*	−1.19	−2.31*	0.11	−0.29	0.58
Salinity	−0.67	−1.60	1.31	−0.06	−0.83	−0.06	−0.80	−1.35	−0.73
NO_3_^−^ (μmol L^−1^)	−0.52	−0.42	−1.82	−0.47	−0.19	−0.47	−2.67*	−0.62	−2.32*
TN (μmol L^−1^)	0.49	0.12	2.21*	0.39	−0.09	0.39	2.91**	0.88	2.39*
NO_2_^−^ (μmol L^−1^)	1.20	2.35*	−1.51	−0.15	1.06	−0.15	−1.02	0.61	−1.17
SiO_3_^2−^ (μmol L^−1^)	1.41	1.60	0.22	2.52*	2.66*	2.52*	−2.03*	−2.17*	−2.49*
T (°C)	−1.71	−3.78**	0.80	−2.48*	−2.99**	−2.48*	1.92	−0.37	2.56*
NH_4_^+^ (μmol L^−1^)	−1.99	−2.11*	−1.78	−2.28	−1.98	−2.28*	−1.64	−1.81	−0.77

a T, temperature; SiO_3_^2−^, silicate; PO_4_^3−^, phosphate; TN, total nitrogen; NO_3_^−^, nitrate; NO_2_^−^, nitrite; NH_4_^+^, ammonium; chlorophyll *a*, the measured chlorophyll *a* concentration. The numbers are *t* values. Asterisks represent significance levels at *P < *0.05 (*) or *P < *0.01 (**).

### Environmental breadth of seawater chlorophyll *a* in both PAB and FLB.

A multiple regression analysis on the distance matrices (MRM) revealed that pure environmental variables explained 4.1% of the particle-associated bacterial community compositions (PACCs) and 20.6% of the free-living bacterial community compositions (FLCCs) (Table S2). The geographical distances between sampling sites also displayed significant correlations with both PACCs and FLCCs, and the effect of pure geographical distance was much higher on PACCs than on FLCCs (Table S2). The changed rate (i.e., community composition dissimilarity per 1,000 km) was 4.41% for PAB (Fig. S4A) and 3.70% for FLB (Fig. S4B).

**FIG 3:**
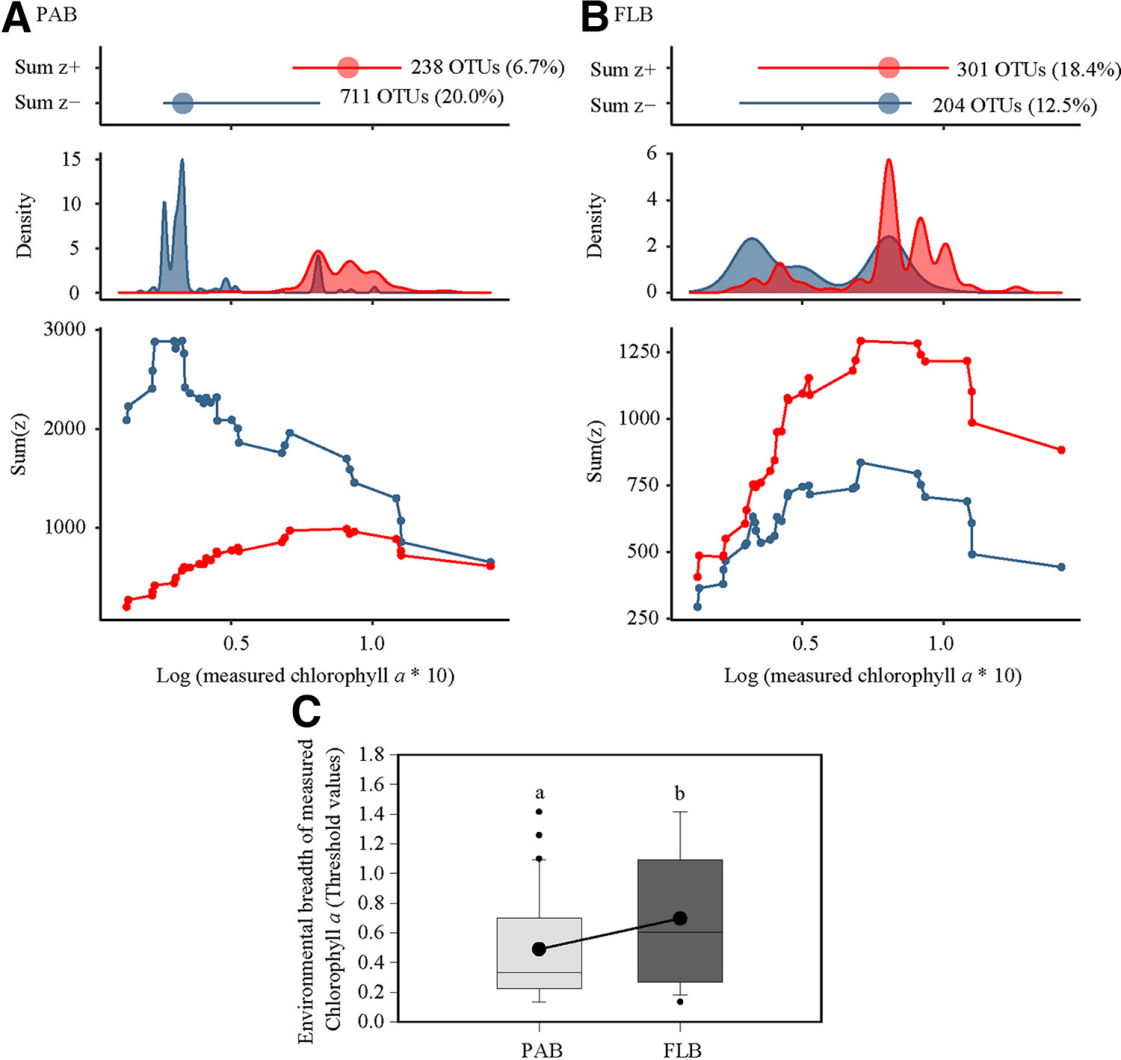
Threshold indicator taxa analysis of the change points of both particle-associated bacteria (PAB) (A) and free-living bacteria (FLB) (B) in response to the measured chlorophyll *a* gradient as well as their overall niche breadth of the measured chlorophyll *a* (C). Red and dark blue symbols and areas show the magnitude of the summed *z* scores of increasing (*z*+) or decreasing (*z*−) taxa with an increasing chlorophyll *a* gradient. The peaks in the values indicate points along the measured chlorophyll *a* gradient that produce large amounts of change in the community structure. The number of OTUs that significantly increased (*z*+) or decreased (*z*−) with an increasing chlorophyll *a* value is also shown in the figure. Significant (*P < *0.05) differences among groups are indicated by different alphabetic letters above the bars.

**FIG 4:**
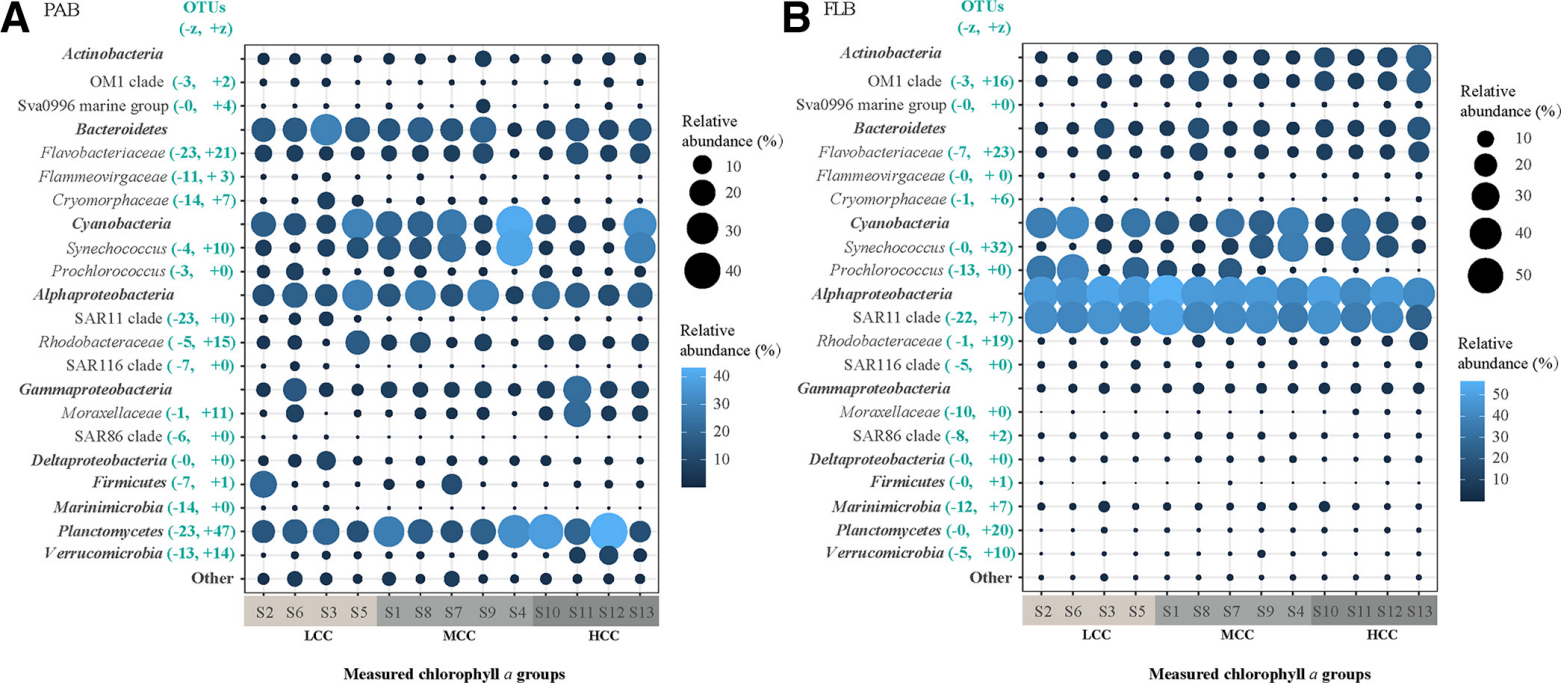
The dominant taxon (phyla, subphyla, clades, or lineages) distributions of both particle-associated bacteria (PAB)(A) and free-living bacteria (FLB) (B), according to different measured chlorophyll *a* groups. LCC, low chlorophyll *a* concentrations (chlorophyll *a *< 0.2 μg/L); MCC, medium chlorophyll *a* concentrations (0.2 μg/L < chlorophyll *a *< 0.5 μg/L); HCC, high chlorophyll *a* concentrations (chlorophyll *a *> 0.5 μg/L). In each clade or lineage, the number of OTUs that significantly increased (*z*+) or decreased (*z*−) with an increasing chlorophyll *a* concentration is also shown in the figure.

Among the investigated environmental variables, the measured chlorophyll *a* was found to be the best predictor of both PACCs and FLCCs, as revealed by partial Mantel tests (Table 2). We further evaluated the positive and negative niche thresholds for both PACCs and FLCCs in response to the changes of the chlorophyll *a* concentration using TITAN. Notably, the particle-associated bacteria exhibited a lower environmental threshold for chlorophyll *a* concentrations (the highest density of *z*− was about 0.2 μg/L of chlorophyll *a*, but *z*+ was approximately 0.5 μg/L) than did the free-living bacteria (the highest density of both *z*− and *z*+ were approximately 0.5 μg/L of chlorophyll *a*) (Fig. 3). We observed that across chlorophyll *a* concentrations, the free-living bacteria had more OTUs at higher chlorophyll *a* concentrations, but, in contrast, the particle-associated bacteria had more OTUs with relative abundances that decreased significantly with an increasing chlorophyll *a* concentration (Fig. 3). Further, we found that the decreasing (*z*−) OTUs with the chlorophyll *a* concentration in both PAB and FLB were primarily affiliated with *Prochlorococcus*, the SAR11 clade, the SAR116 clade, and the SAR86 clade, whereas the increasing (*z*+) OTUs with the chlorophyll *a* concentration mainly belonged to *Synechococcus*, *Rhodobacteraceae*, and *Flavobacteriaceae* (Fig. 4).

**TABLE 2:** Spearman’s correlations (*r* values) between transformed environmental variables and particle-associated bacterial (PAB) and free-living bacterial (FLB) community compositions, as determined via partial Mantel tests^*a*^

Index	PAB		FLB	
	*r*	*P*	*r*	*P*
Chlorophyll *a* (μg/L)	0.253	0.01**	0.308	0.01**
PO_4_^3−^ (μmol L^−1^)	0.048	0.198	0.187	0.01**
Salinity	0.204	0.01**	0.234	0.01**
NO_3_^−^ (μmol L^−1^)	0.162	0.01**	0.104	0.05*
TN (μmol L^−1^)	0.136	0.01**	0.044	0.174
NO_2_^−^ (μmol L^−1^)	−0.009	0.521	−0.067	0.897
SiO_3_^2−^ (μmol L^−1^)	0.219	0.01**	0.206	0.01**
T (°C)	0.092	0.044*	0.197	0.01**
NH_4_^+^ (μmol L^−1^)	−0.077	0.963	0.079	0.065

a *, *P *< 0.05; **, *P *< 0.01.

### Beta diversity patterns of both PAB and FLB.

**FIG 5:**
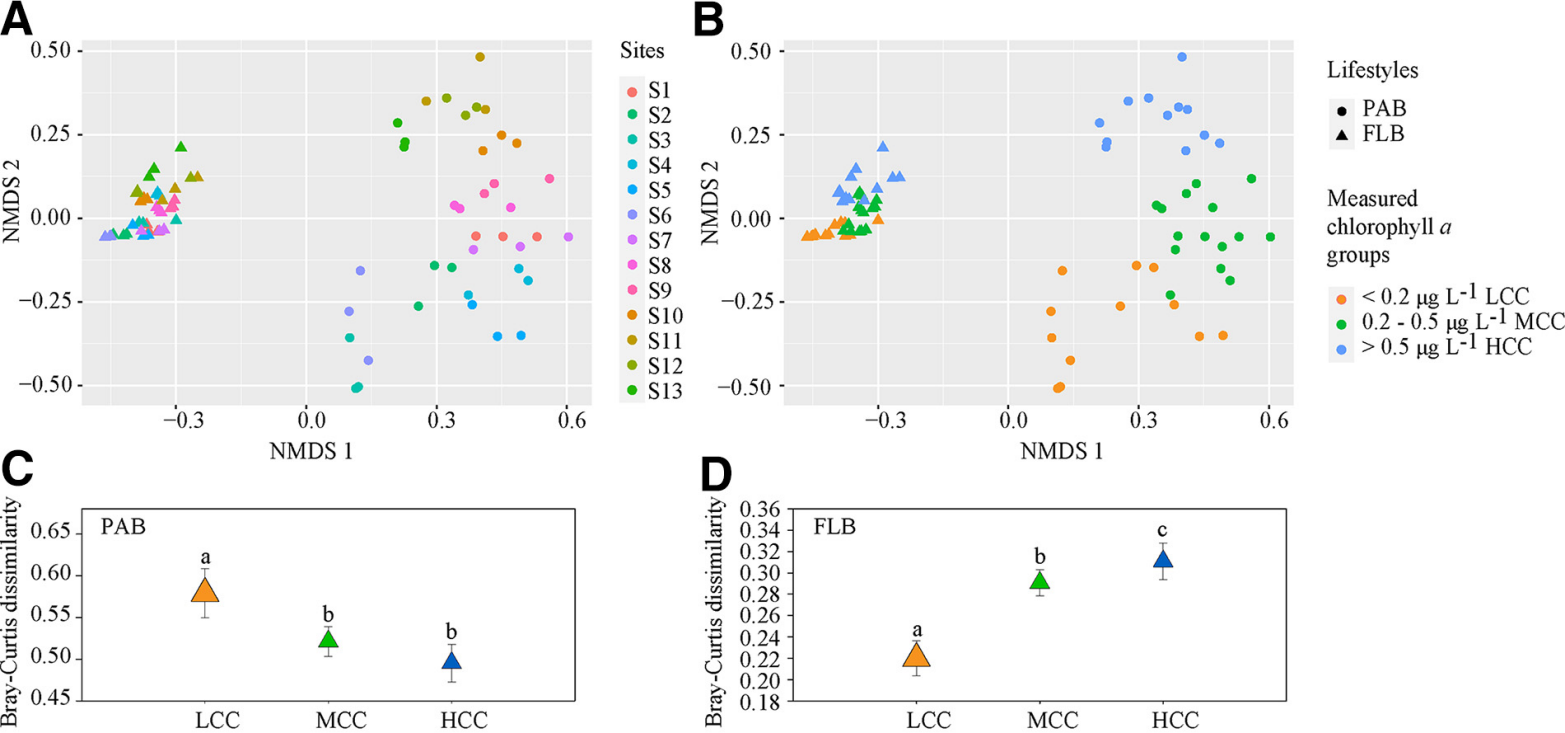
The nonmetric multidimensional scaling (NMDS) plots derived from the Bray-Curtis dissimilarities of particle-associated bacteria (PAB) and free-living bacteria (FLB) across different sampling sites (A) or in different measured chlorophyll *a* groups (B) as well as the beta diversity of both PAB (C) and FLB (D) in different chlorophyll *a* groups. LCC, low chlorophyll *a* concentrations (chlorophyll *a *< 0.2 μg/L); MCC, medium chlorophyll *a* concentrations (0.2 μg/L < chlorophyll *a *< 0.5 μg/L); and HCC, high chlorophyll *a* concentrations (chlorophyll *a *> 0.5 μg/L). Significant (*P < *0.05) differences among groups are indicated by different alphabetic letters above the bars.

The nonmetric multidimensional scaling (NMDS) based on the Bray-Curtis dissimilarities revealed that a significant divergence of community structure was observed between PAB and FLB (PERMANOVA: *F* = 52.14, *P < *0.001) (Fig. 5A and B). In addition, significantly higher beta diversity was observed in PAB than in FLB (permutation *t* test: *t* = 43.781, *P < *0.01) (Fig. S5). We further observed that neither the PAB nor the FLB community structure exhibited obvious spatial patterns (Fig. 5A), but they were arranged according to their chlorophyll *a* concentrations as low chlorophyll *a* concentrations (LCC; chlorophyll *a *< 0.2 μg/L), medium chlorophyll *a* concentrations (MCC; 0.2 μg/L < chlorophyll *a *< 0.5 μg/L), and high chlorophyll *a* concentrations (HCC; chlorophyll *a *> 0.5 μg/L) (Fig. 5B). Significant differences in community structure were found among these three groups of LCC, MCC, and HCC for both PAB and FLB (PERMANOVA: *F* = 7.754, *P < *0.001 for PAB and F = 12.340, *P < *0.001 for FLB) (Table 3) as well as between all pairwise groups (PERMANOVA: F > 5.950, *P < *0.01 in all cases for PAB, F > 5.396, *P < *0.01 in all cases for FLB) (Table 3). The beta diversity was also significantly different within the three groups of LCC, MCC, and HCC for both PAB (permutation ANOVA: F = 6.604, *P < *0.01) (Fig. 5C) and FLB (permutation ANOVA: F = 11.602, *P < *0.01) (Fig. 5D). For FLB, we observed a higher chlorophyll *a* concentration that was related with a higher beta diversity of free-living bacterial communities (pairwise permutation *t* tests: *P < *0.05) (Fig. 5D), but, for PAB, a higher chlorophyll *a* concentration was linked to a lower beta diversity (pairwise permutation *t* tests: *P < *0.05) (Fig. 5C). Of the chlorophyll *a* groups (LCC, MCC, and HCC), the beta diversity between each chlorophyll *a* group and the other chlorophyll *a* groups was more than 1.3 times of that observed within the chlorophyll *a* groups, and it had significant differences for both PAB (permutation ANOVA: F = 23.207, *P < *0.01) (Fig. S6A) and FLB (permutation ANOVA: F = 31.057, *P < *0.01) (Fig. S6B). However, compared with the LCC and HCC bacterial communities, the MCC bacterial community had significantly lower differences, compared to the other chlorophyll *a* groups for both PAB (pairwise permutation *t* test: *t* < −4.506, *P < *0.01) (Fig. S6A) and FLB (pairwise permutation *t* test: *t* < −5.297, *P < *0.01) (Fig. S6B).

**TABLE 3:** Pairwise PERMANOVA of bacterial community structure, based on Bray-Curtis dissimilarity^*a*^

Groups	PAB		FLB	
	*F*	*P*	*F*	*P*
Whole	7.754	0.001**	12.34	0.001**
LCC versus MCC	5.950	0.003**	10.600	0.003**
LCC versus HCC	8.859	0.003**	21.776	0.003**
MCC versus HCC	7.801	0.003**	5.396	0.006**

a PAB, particle-associated bacteria; FLB, free-living bacteria. LCC, low chlorophyll *a* concentrations (chlorophyll *a* < 0.2 μg/L); MCC, medium chlorophyll *a* concentrations (0.2 μg/L < chlorophyll *a* < 0.5 μg/L); HCC, high chlorophyll *a* concentrations (chlorophyll *a* > 0.5 μg/L). *, *P *< 0.05; **, *P *< 0.01.

### Community assembly processes underlying the biogeographical and biodiversity patterns of both PAB and FLB.

Significantly positive relationships between the OTU phylogenetic distances and the niche distances (phylogenetic signals) were observed across relatively short phylogenetic distances (less than 10% of the maximum phylogenetic distance) for both PAB and FLB (Fig. S7A and B). These relationships suggest that the closely related OTUs of both PAB and FLB were niche conservatism in that closely related OTUs were more similar to each other, in terms of niche, than were distant relatives. When we detected the phylogenetic distribution of the top 30 most abundant taxa in both PAB and FLB as well as their environmental preferences, we found that most of the closely related taxa (OTUs) in both PAB (e.g., *Synechococcus*: OTU-1 and OTU-4541; *Planctomycetaceae*: OTU-4212 and OTU-80; *Tropicibacter*: OTU-24 and OTU-27; NS2b marine group: OTU-12 and OTU-619) and FLB (e.g., *Prochlorococcus*: OTU-492 and OTU-17; *Synechococcus*, OTU-1831 and OTU-2344; SAR11: OTU-2093, OTU-235, OTU-23, and OTU-5049; NS4 marine group: OTU-9 and OTU-33) had similar environmental preferences (Fig. S8A and B).

**FIG 6:**
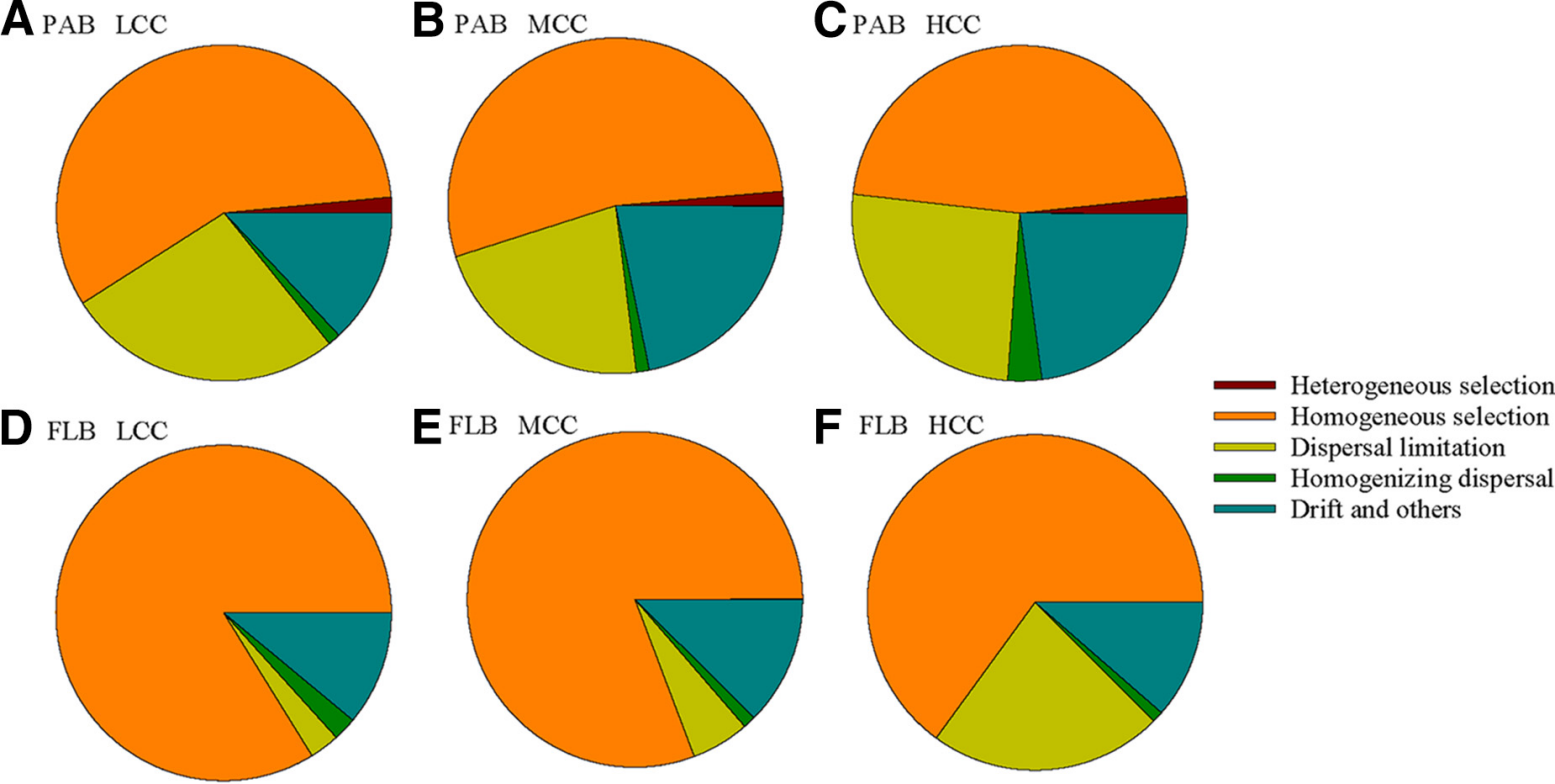
The relative importance of community assembly processes of particle-associated bacteria (PAB) (A–C) and free-living bacteria (FLB) (D–F) within groups of different chlorophyll *a* concentrations. The community assembly processes include homogeneous and heterogeneous selection, dispersal limitations, homogenizing dispersal, and the drift and other fractions. LCC, low chlorophyll *a* concentrations (chlorophyll *a *< 0.2 μg/L); MCC, medium chlorophyll *a* concentrations (0.2 μg/L < chlorophyll *a *< 0.5 μg/L); and HCC, high chlorophyll *a* concentrations (chlorophyll *a *> 0.5 μg/L).

The community assembly processes were further analyzed. The results showed that deterministic assembly, primarily homogeneous selection, dominated the planktonic bacterial community assembly and contributed a larger fraction to FLB (76.7%) than to PAB (49.5%) (permutation *t* test: *t* = 55.976, *P < *0.001) (Fig. S9). In contrast, dispersal limitation (permutation *t* test: *t* = 34.526, *P < *0.001) and drift (permutation *t* test: *t* = 15.478, *P < *0.001) influenced the community assembly of PAB more than that of FLB (Fig. S9). Within the three groups of chlorophyll *a* concentration (LCC, MCC, and HCC), we found higher relative contributions of drift processes in the groups with higher chlorophyll *a* concentrations in the PAB community assembly (permutation ANOVA: *F* = 29.365, *P < *0.01) (Fig. 6A–C). However, the FLB community assembly exhibited a lower relative contribution of homogeneous selection (permutation ANOVA: *F* = 142.47, *P < *0.01) but a higher relative contribution of dispersal limitation (permutation ANOVA: *F* = 141.34, *P < *0.01) in the groups with higher chlorophyll *a* concentrations (Fig. 6D–F).

Compared with the bacterial community assembly processes within the groups of LCC, MCC, and HCC, we found that the assembly processes between each group and the other groups of LCC, MCC, and HCC were composed of weaker effects of homogeneous selection and homogenizing dispersal, stronger effects of dispersal limitation, and no obvious effects of heterogeneous selection and drift (Fig. 6; Fig. S10). In addition, compared with the LCC and MCC bacterial communities, the HCC bacterial community had significantly weaker effects of homogeneous selection (PAB pairwise permutation *t* test: *t* < −4.968, *P < *0.01; FLB pairwise permutation *t* test: *t* < −6.516, *P < *0.01), a significantly stronger effect of dispersal limitation (PAB pairwise permutation *t* test: *t* > 2.167, *P < *0.05; FLB pairwise permutation *t* test: *t* > 7.468, *P < *0.01), but no significant effects of drift (PAB pairwise permutation *t* test: *t* < 0.744, *P > *0.05; FLB pairwise permutation *t* test: *t* < 1.005, *P > *0.05) with bacterial communities in the other chlorophyll *a* groups (Fig. S10).

## DISCUSSION

In this study, we investigated the biogeographical and biodiversity patterns of marine planktonic bacteria across a broad chlorophyll *a* gradient spanning from the South China Sea across the Gulf of Bengal to the northern Arabian Sea. We found that the seawater chlorophyll *a* concentration was significantly correlated with the alpha diversity patterns of both PAB and FLB. However, FLB and PAB exhibited contrasting alpha diversity patterns along the broad chlorophyll *a* gradient, with positive linear relationship for FLB but a negative correlation for PAB. Compared with FLB, PAB exhibited a narrower niche breadth of diversity patterns across the broad chlorophyll *a* gradient, with far fewer bacterial taxa being favored at higher chlorophyll *a* concentrations. Moreover, we found that the community assemblies of planktonic PAB and FLB were determined by the different combinations of selection, dispersal, and stochastic drift fractions, which contributed to the observed biogeographical and biodiversity patterns of the PAB and FLB spanning from the South China Sea, across the Gulf of Bengal, to the northern Arabian Sea.

### The biogeographical pattern of marine planktonic bacteria complied with the scenario of homogeneous selection and selected taxa by the environmental factor of chlorophyll *a* concentration.

Dispersal is important in the emergence and maintenance of plankton biogeography (23, 25, 39, 46, 47). In this study, sampling sites from the South China Sea, across the Gulf of Bengal, to the northern Arabian Sea might be connected by ocean currents, such as the winter western Indian Coastal Current, the Northeast Indian Monsoon Current, and the surface circulation of the South China Sea (48–50). Because marine plankton bacteria are unable to swim against currents, their (plankton bacteria) biogeography is shaped by current systems, as demonstrated by a recent study that analyzed the metagenomes of plankton communities that were sampled across global oceans during the Tara Oceans expedition (47). In this study, we also observed that the current-related geographical distances significantly contributed to planktonic bacterial distributions; however, the effect of distance-related dispersal was not high enough to result in obvious dispersal limitation and current-related spatial patterns (Fig. 5A and B). In contrast, similar compositions were observed in geographically distant sampling sites (e.g., sites S1 and S7) with similar seawater chlorophyll *a* concentrations (Fig. 5A and B). Therefore, strong dispersal limitation was impossible within the interconnected sampling sites that were included in this study. Significant heterogeneity of the bacterial community compositions of both PAB and FLB was found, and it was significantly related with the seawater chlorophyll *a* concentration, indicating that the marine planktonic bacterial community assembly complied with the scenario of homogeneous selection (i.e., the selection of taxa by the environmental conditions). In this study, the rates of bacterial dispersal might be moderate for most bacterial taxa, and this supports homogeneous selection in the local habitats by providing sufficient bacterial taxa from the broad species pool (38, 46, 51). Our findings were supported by recent studies that were conducted both in the Pacific Ocean over a transect of 12,400 km between subantarctic and subarctic regions (25) and in tropical and subtropical ocean areas during circumglobal expeditions (23), suggesting that homogeneous selection has a stronger effect on global planktonic bacterial community assemblages than do dispersal limitation (low dispersal) and homogenizing dispersal (high dispersal) (23, 25). Nevertheless, different from the results of our study, both of these two studies (23, 25) indicated that instead of the chlorophyll *a* concentration, the seawater temperature is important for the significance of the environmental selection for the planktonic bacterial community assembly (23, 25). The inconsistent findings might be due to the fact that, compared with the temperature differences in the previous studies (from 0 to 28°C), the temperature span (from 0 to 5°C) in this study was not broad enough to impose significant environmental selection on the bacterial community assembly (23, 25, 52).

The high efficiency of homogeneous selection in the bacterial community assembly was probably due to the high sensitivity of bacteria to seawater chlorophyll *a* (18, 53). We also observed that the seawater chlorophyll *a* concentration acted as a key environmental factor relating to the biogeographical patterns of both PAB and FLB. Our results are consistent with those of previous studies, which indicated that the seawater chlorophyll *a* concentration, expressed in terms of the resource supply, was the principal factor relating to local marine species diversity (4, 18). We further found that both the PAB and FLB community compositions diverged, according to chlorophyll *a* concentrations that were categorized into low (<0.2 μg/L), medium (0.2 to 0.5 μg/L), and high (>0.5 μg/L) groups. The decreasing (*z*−) taxa with an increasing chlorophyll *a* concentration of both PAB and FLB were primarily affiliated with *Prochlorococcus*, the SAR11 clade, the SAR116 clade, and the SAR86 clade. These organisms have been well-documented to be optimized for an oligotrophic lifestyle with minimalist genomes, small cell sizes, and specialized resource acquisition abilities, such as photosynthesis or phototrophy by proteorhodopsins (54–56). However, the increasing (z+) taxa with an increasing chlorophyll *a* concentration of both PAB and FLB mainly belonged to *Synechococcus*, *Rhodobacteraceae*, and *Flavobacteriaceae*, the relative abundances of which have been demonstrated to be positively correlated with seawater nutrient loads in recent studies (57, 58).

### FLB and PAB exhibited contrasting diversity patterns across a broad chlorophyll *a* gradient.

In this study, the taxonomic diversity (OTU richness), phylogenetic diversity, and Shannon index, which can minimize the influences of the species abundance distribution (59), were calculated to assess the plankton bacterial alpha diversity. We found that these three indexes of alpha diversity exhibited similar patterns along the broad chlorophyll *a* gradient in this study. For the free-living bacteria, these three alpha diversity indexes all displayed a regular positive linear relationship with the seawater chlorophyll *a* concentration, and these relationships were qualitatively similar to those that were observed for eukaryotes (7, 14, 60). The finding observed in the chlorophyll *a* and FLB alpha diversity relationships is in line with the concept of “complementarity”, which are related to species niche differentiation and, thus, functional complementarity to utilize resources more completely (9, 10, 60). However, PAB and the total planktonic bacteria exhibited a contrasting diversity pattern to FLB across the chlorophyll *a* gradient, showing negative linear correlations between the chlorophyll *a* concentration and the PAB taxonomic and phylogenetic diversities as well as the Shannon index.

In marine water columns, PAB and FLB generally coexist with obvious niche differentiation (28, 29). Compared to the state of FLB, PAB occur in or on the substratum that is composed mostly of organic matter, such as aggregates and organisms (34, 61). The niches of PAB can range from sparse to dense and from monospecific to highly diverse (34, 61). Therefore, although there might be a frequent exchange between PAB and FLB in the local communities through attachment-detachment cycles (34), we found significantly different community compositions for PAB and FLB. The typical algae-associated bacteria, including *Flavobacteriaceae*, *Planctomycetes*, and *Verrucomicrobia* were observed to have obviously increased in their relative abundance of PAB, reflecting a strong species sorting effect imposed by the substratum of PAB (44, 62, 63). The hosts of PAB might attract specific bacteria groups from the ambient seawater through chemical and physical stimuli (64), and some of the bacteria that were selected by their hosts have been found to play major roles in the marine carbon cycle by utilizing organic carbon sources that are released from the host cells (44). In addition, PAB have been found to be more densely packed by 1 to 2 orders of magnitude, having higher growth, higher production, and more intense lateral gene transfer than FLB (34). This might explain why PAB displayed a significantly higher alpha diversity than did FLB and determined the overall biogeography pattern of the total bacteria. However, it must be kept in mind that although a significantly higher bacterial alpha diversity was observed for PAB, PAB generally have abundances of 10^2^ cells/mL in surface seawaters, whereas FLB typically exhibit abundances of 10^4^ to 10^5^ cells/mL (65, 66). Increases in FLB alpha diversity may enhance remineralization and thereby increase the availability of nutrients for phytoplankton (58).

Our previous studies reported that the highest chlorophyll *a* concentration that was found in the northern Arabian Sea was caused by the wintertime dinoflagellate (*Noctiluca scintillans*) blooms (40, 41). Massive blooms of *Noctiluca scintillans* probably substantially simplify the diversity of phytoplankton and zooplankton, partly due to oxygen depletion and intraspecific and interspecific competition (44, 67), which could result in less heterogeneity of organic substratum (e.g., substratum microtopography and substances released by the substratum) for PAB, which may partly explain that the diversity pattern of PAB monotonically declined with the increasing seawater chlorophyll *a* concentration. Concurrently, the analysis of niche thresholds showed that PAB exhibited a narrower chlorophyll *a* niche breadth than did FLB, with far fewer bacterial taxa being favored by a high chlorophyll *a* concentration.

### A higher chlorophyll *a* concentration is linked to the stronger stochastic drift processes and lower beta diversity of PAB but is also related to the weakened homogeneous selection, enhanced dispersal limitation, and increased beta diversity of FLB.

The seawater chlorophyll *a* concentration not only had strong impacts on the alpha diversity patterns of marine planktonic bacteria but also may significantly change the bacterial beta diversity (18). We observed that a higher chlorophyll *a* concentration reduced the beta diversity of PAB but increased the beta diversity of FLB. The change in beta diversity might be the result of community assembly mechanisms involving purely deterministic processes, purely stochastic processes, and the interaction between deterministic and stochastic processes when stochastic variation in the history of colonization results in deterministic priority effects that vary across habitats (37). These factors can be integrated into community assembly mechanisms that include homogeneous and heterogeneous selection, dispersal limitations, homogenizing dispersal, and stochastic drift fractions (35, 36). In our study, the higher beta diversity of PAB than FLB might relate to the enhanced stochastic processes, including ecological drift and dispersal limitation. Moreover, the reduced beta diversity of PAB at higher chlorophyll *a* concentrations might reflect the enhanced effect of stochastic ecological drift, whereas the increased beta diversity of FLB under higher chlorophyll *a* concentrations might reflect the weakened roles of homogeneous selection and enhanced dispersal limitations.

The dispersal rates of PAB not only are dependent on ocean currents but also are affected by the dispersal capabilities of their hosts, such as aggregates, phytoplankton, or zooplankton (28, 29). Because of the random dispersal principle, the more abundant and the smaller cell size microorganisms are expected to disperse more thoroughly than are the less abundant and larger ones (68). Therefore, compared with FLB, the larger and less abundant PAB might get a stronger effect of dispersal limitation (25). In our study, both an MRM analysis and the iCAMP framework revealed that dispersal limitation played a more important role in determining the community assembly of PAB than that of FLB, indicating that PAB have a higher dispersal limitation than do FLB. Similar findings were observed in previous studies (19, 25, 31), and it was further confirmed by the higher change rate (i.e., community composition dissimilarity per 1,000 km) of PAB than of FLB. Similar patterns between bacterial community similarities and geographical distances within 6,000 km were also observed in recent studies that were conducted both in the Atlantic Ocean (26) and in the Pacific Ocean (25). However, both studies showed that the change rates of both PAB and FLB were above 5.0% community composition dissimilarity per 1,000 km (25, 26), which was slightly higher than those that were observed in our study. These inconsistent results might be partly due to the differences in sampling depth and ocean basins. It shows that bacterial samples were collected over the whole epipelagic (depth from 0 to 100 m) in the studies of the Atlantic Ocean and the Pacific Ocean (25, 26), whereas, in our study, the samples were collected from the near-surface seawater (depth < 0.5 m) spanning from the South China Sea to the northern Arabian Sea.

This enhanced dispersal limitation, in combination with the increased ecological drift, might contribute to the higher beta diversity of PAB than of FLB (69). In contrast to PAB, higher homogeneous selection was observed, and it dominated the community assembly of FLB, indicating that FLB have more homogeneous habitats than do PAB (29, 32). Our study was consistent with a recent study on microbial biogeographic patterns spanning from subantarctic to subarctic regions in the Pacific Ocean (25), which also demonstrate a stronger role of homogeneous selection in determining free-living (<0.2 to 3 μm) bacterial community assemblies than in other 3 to 8 μm and > 8 μm bacterial communities in the near-surface seawater (25). Our previous studies reported that the high seawater chlorophyll *a* concentration was caused by the wintertime *Noctiluca scintillans* blooms in the northern Arabian Sea (40, 41). These blooms are triggered by the nutrient transports by convective mixing in response to ocean surface cooling, and they are maintained by the continuous nutrient supply by the horizontal and vertical advection caused by mesoscale eddies (70, 71). The nutrient supply lowers the *Noctiluca scintillans* growth limitation and results in an increase in the seawater chlorophyll *a* concentration (41–43). For marine planktonic bacteria, the dispersal rate of FLB is primarily dependent on ocean currents. The mesoscale eddies in the northern Arabian Sea most likely inhibited the dispersal efficiency of FLB across the sampling habitats (71–73) and increased the relative importance of dispersal limitation in determining the FLB community assembly, thereby causing a higher beta diversity of FLB in habitats with high chlorophyll *a* concentrations. The increased beta diversity of FLB might also relate to the weakened homogeneous selection. The existing studies suggest that a lower chlorophyll *a* concentration (primary oligotrophic water mass) is more likely to have stronger environmental selection in sorting a subset of ecologically similar taxa that are capable of thriving under oligotrophic conditions (18). We observed that closely related taxa (OTUs) with high relative abundances in FLB (e.g., *Prochlorococcus*: OTU-492 and OTU-17, SAR11: OTU-2093, OTU-235, OTU-23, and OTU-5049) had similar environmental preferences for oligotrophic conditions. These bacteria have specialized resource acquisition abilities, such as photosynthesis or phototrophy by proteorhodopsins (54–56). In addition, the convective mixing and eddy flows might also weaken the dispersal of PAB across sampling sites (31). Thus, the high abundance of *Noctiluca scintillans* (probably the main host of PAB) in the northern Arabian Sea did not decrease the relative importance of dispersal limitation in determining the PAB community assembly but increased the role of ecological drift in shaping the PAB community assembly, thereby resulting in a lower beta diversity of PAB at higher chlorophyll *a* concentrations.

In summary, our biogeographical study demonstrated that primary productivity, indicated as the chlorophyll *a* concentration, correlated with the alpha diversity patterns of marine planktonic bacteria. However, FLB and PAB displayed contrasting alpha diversity and primary productivity relationships with a positive linear correlation for FLB but a negative correlation for PAB. Moreover, the biogeographical patterns of planktonic PAB and FLB were determined by the different combinations of selection, dispersal, and stochastic drift fractions, which contributed to the observed beta diversity patterns of PAB and FLB. The changes in marine planktonic PAB and FLB biodiversity might have different trophic cascade effects on other prokaryotes and eukaryotes and could change ecosystem functioning, such as the ecosystem stability, primary productivity, mineralization of organic matter, nitrification, ammonization, and production and consumption of methane. This coupling between planktonic PAB and FLB biodiversity and ecosystem functioning might provide clues to take them into account in future studies that aim to predict the impacts of changed biodiversity on ecosystem functioning, particularly in the current scenario of climate change, which is supposed to not only increase the frequency of eutrophication but also have impacts on planktonic biodiversity. However, because FLB and PAB might display contrasting relationships (e.g., positive, negative, linear, or nonlinear) with ecosystem functioning, PAB and FLB might need to be considered independently in ecosystem predictive models that aim to assess marine ecosystem functioning under future scenarios of eutrophication.

## MATERIALS AND METHODS

### Sample collection and measurement of environmental factors.

From January to February of 2018, a total of 78 water samples were collected on board the “Experiment 3” scientific research ship from 13 stations (Fig. 1) during the China-Pakistan Joint Expedition, which spanned from the South China Sea, across the Gulf of Bengal, to the northern Arabian Sea. These 13 stations covered a broad range of the annual chlorophyll *a* concentration (i.e., 0.12 to 2.89 μg/L), which was obtained before the sampling time from the NASA MODIS OceanColor website. The map of the sampling sites was generated based on an open-access Google satellite map using the ggmap package (https://github.com/dkahle/ggmap) in the R statistical environment (74) (Fig. 1). At each station, water samples (1 to 2 L water) were collected from surface waters (the top 50 cm) at 3 replicate sites. At each sampling point, we separated samples into particle-associated and free-living bacterial subsamples by filtering them through 3 μm pore-size Isopore filters (Millipore, Billerica, MA, USA) and then through 0.2 μm pore-size Isopore filters (Millipore, Billerica, MA, USA), respectively (61). The filters were stored at −80°C for further analyses. The salinity and temperature (T) were measured by the ship-equipped CTD (Sea-bird) during the cruise. At each sampling point, a 1 L water sample was collected from the surface waters for nutrient analyses. Phosphorus (PO_4_^3−^), nitrate (NO_3_^−^), nitrite (NO_2_^−^), ammonia (NH_4_^+^), and silicate (SiO_3_^2−^) were analyzed using a flow injection analyzer (QuichChem8500, Lachat Inc., Loveland, CO, USA) (75). The total nitrogen (TN) was measured by the flow injection analyzer after being oxidized with persulphate in an alkaline medium (75). The water chlorophyll *a* concentration was measured using a Turner Designs fluorometer 10-AU, following the method of Parsons et al. (76). The data of the annual chlorophyll *a*, annual temperature, and annual POC were obtained before the sampling time, directly from the NASA MODIS OceanColor website (http://oceancolor.gsfc.nasa.gov/).

### DNA extraction, amplification, sequencing, and data processing.

DNA extractions were performed using a PowerWater DNA Isolation Kit (MoBio Laboratories, Carlsbad, CA, USA), which is now available as the DNeasy PowerWater DNA Isolation Kit (Qiagen, Hilden, Germany). Bacterial diversity was assessed via the amplicon sequencing of the V4 region of the 16S rRNA gene with an Illumina HiSeq PE250 platform, using the primers 515F- (5′-GTGCCAGCMGCCGCGGTAA-3′) and 806R (3′-GGACTACHVGGGTWTCTAAT-5′). Briefly, a specific 12-mer tag was added to the 5′ end of each primer for each DNA sample to distinguish the samples in one Illumina sequencing run. In each sample, 3 replicates were PCR amplified in a 50 μL reaction mixture, including 25 μL 2× PCR Premix Taq, 10 mM each primer, 60 ng of genomic DNA, and 20 μL of nuclease-free water. The PCR conditions included 94°C for 5 min, followed by 30 cycles of denaturation at 94°C for 30 s, annealing at 52°C for 30 s, extension at 72°C for 30 s, and a final extension at 72°C for 10 min. Finally, the amplicons were sequenced using an Illumina HiSeq PE250 platform at Novogene Bioinformatics Technology Co., Ltd. (Beijing, China). The sequencing data that were generated in this study were deposited into the National Center for Biotechnology Information (NCBI) Sequence Read Archive (SRA) (https://www.ncbi.nlm.nih.gov/sra) with the accession number PRJNA853635 (TaxID: 410658).

The raw reads of the 16S rRNA gene sequences were analyzed using the mothur software package (v.1.36.1, http://www.mothur.org) (77). Briefly, we (i) combined the two sets of raw reads; (ii) eliminated the sequences with ambiguous base pairs and sequences with lengths that were inconsistent with the target region; (iii) merged duplicate sequences; (iv) preclustered and aligned the unique sequences to the SILVA v132 databases (78); (5) removed the chimeric sequences via the UCHIME algorithm and classified the clean sequences using the SILVA v132 databases at the recommended bootstrap threshold of 80%; (vi) removed the sequences of chloroplasts, mitochondria, archaea and eukaryotes; (vii) clustered the high-quality sequences into operational taxonomic units at a 97% similarity level (OTUs_0.03_); (viii) excluded OTUs occurring in fewer than 3 samples from the following analyses (e.g., bacterial diversity, niche thresholds, community structure, and community assembly) to minimize bias caused by sequencing depth (79); and (ix), in the following analyses, rarefied the sequence number in each sample to the same depth (21,000 sequences per sample) to reduce errors and allow for the performance of a relatively fair comparison among all samples (79).

### Diversity estimation and its niche thresholds.

The marine plankton bacterial alpha diversity was assessed by three indexes, including the richness of the OTUs_0.03_, Faith’s phylogenetic diversity, and the Shannon diversity. The richness of the OTUs_0.03_ and Faith’s phylogenetic diversity represented taxonomic and phylogenetic diversity, respectively. In addition, the widely used Shannon index was also calculated to minimize the influence of the species abundance distribution (59). The alpha diversity was calculated using the R package of vegan. To identify the key environmental factors in shaping the changes in the bacterial alpha diversity, generalized linear models (GLMs) were constructed via the stats package in R. Based on the Bray-Curtis dissimilarity, the bacterial beta diversity was assessed in groups of both PAB and FLB as well as in groups with different chlorophyll *a* concentrations (LCC, MCC, and HCC). It was further visualized using nonmetric multidimensional scaling (NMDS), which was implemented using the vegan package in R. For the LCC, MCC, and HCC groups, the bacterial beta diversity was also calculated between groups of different chlorophyll *a* concentrations (LCC, MCC, and HCC). To infer the relative importance of environmental drivers and geographical distance in shaping the biogeographical patterns of the PAB and FLB, MRM was performed to partition the variance of the Bray–Curtis dissimilarity into (i) pure environmental variation, (ii) pure geographical variation, (iii) spatially structured environmental variation, and (iv) unexplained variation using the ecodist package in R (80). The pure effect of environment was further tested to find the key environmental variables in determining the PAB and FLB community structure via partial Mantel tests using the mantel.partial command in the vegan package in R. The niche threshold of both PAB and FLB in response to the measured chlorophyll *a* concentration was further calculated via a threshold indicator taxa analysis (TITAN) (81) in the TITAN2 package in R. The TITAN analysis can identify the significantly increasing (*z*+) or declining (*z*−) taxa responses to the chlorophyll *a* changes and track the cumulative number of increasing (sum[*z*+]) and declining (sum[*z*−]) taxa in each community (81). The sum and the density of the increasing (*z*+) or declining (*z*−) taxa in each bacterial community were plotted across the chlorophyll *a* gradient, and the taxonomic information of the indicators was identified. The environmental points with the highest sum or density of the increasing (*z*+) or declining (*z*−) taxa were used as evidence for the positive and negative niche thresholds of both PAB and FLB (81).

### The phylogenetic signal and the community assembly mechanisms.

To evaluate the phylogenetic signal of both marine PAB and FLB, we constructed a Mantel correlogram that was based on the Pearson correlation coefficients between the differences in the phylogenetic distances and environmental traits via the mantel.correlog function in the vegan package in R (82). The significance of the correlations was tested by 1,000 permutations with a progressive Bonferroni correction. In addition, to infer the community assembly mechanisms shaping the plankton bacterial biogeography, we performed community assembly mechanisms by a phylogenetic, bin-based null model analysis (iCAMP) framework that was modified by Ning et al. (35) from a previous framework (83). The iCAMP framework provides an effective and robust tool with which to quantify the relative importance of different ecological processes for each phylogenetic group (bin), rather than for the entire community (35), as the phylogenetic conservation of microbial communities is always observed across relatively short phylogenetic distances (35). Bacterial taxa (OTUs) were divided into different bins, based on their phylogenetic relationships. A mantel test was then used to assess the phylogenetic signal for each individual bin. For each bin, the beta net relatedness index (βNRI) and a taxonomic dissimilarity metric using the Bray-Curtis-based Raup-Crick (RC_bray_) were further calculated (83).

According to Ning et al. (35), a βNRI value of >1.96 or <−1.96 indicates significantly greater or less phylogenetic turnover than expected, respectively, indicating the predominance of deterministic processes (heterogeneous selection and homogeneous selection, respectively). In contrast, |βNRI| ≤ 1.96 indicates that stochastic processes predominate. The contributions of pairwise comparisons with |RC_bray_| > 0.95 indicate homogenizing dispersal or dispersal limitation, whereas those with |RC_bray_| ≤ 0.95 represent the importance of undominated processes (drift and others), which arise when there is a moderate rate of dispersal and the strength of selection is relatively weak (84). Strong selection results in heterogeneous selection or homogeneous selection, and low dispersal rates lead to dispersal limitation, whereas high dispersal rates relate to homogenizing dispersal (84). In this study, the iCAMP framework was performed over the whole bacterial sample set, which included 78 samples (half FLB and half PAB), as these bacterial samples may share the same species pool. We then obtained the relative importance of heterogeneous selection, homogeneous selection, dispersal limitation, homogenizing dispersal, drift, and other factors in homogenous groups of PAB and FLB, in homogenous groups of different chlorophyll *a* concentrations (LCC, MCC, and HCC), and in heterogeneous groups of LCC, MCC, and HCC from the whole result set.

### Statistical analyses.

A principal components analysis (PCA) and Spearman’s ρ rank correlation were used to depict the relationships among all of the investigated environmental variables using the vegan and corrgram packages in, respectively. To test the significant differences between bacterial community structures in groups of different chlorophyll *a* concentrations (LCC, MCC, and HCC), a permutational multivariate analysis of variance using distance matrices (PERMANOVA) was carried out, based on the Bray-Curtis dissimilarity, using the vegan package in R. Spearman’s ρ rank correlations were also calculated so as to find the environmental preferences of the top 30 most abundant taxa of the entire sample set. By using FastTree (85), the phylogenetic relationship of the top 30 most abundant taxa was further constructed. A permutation analysis of variance (permutation ANOVA), which was followed by a pairwise permutation *t* test and a permutation Student’s *t* test, was performed to analyze the significant differences of the beta diversity and community assembly processes in groups of PAB and FLB as well as within and between groups of different chlorophyll *a* concentrations, respectively, using the RVAideMemoire package in R (86).
