# Supplementary material for: Biogeographical and Biodiversity Patterns of Marine Planktonic Bacteria Spanning from the South China Sea across the Gulf of Bengal to the Northern Arabian Sea
Source: Microbiol Spectr. 2023 Apr 26;11(3):e00398-23. doi: 10.1128/spectrum.00398-23 (PMC10269852; doi:10.1128/spectrum.00398-23)
Supplement: Supplemental file 1 — Supplemental material. Download spectrum.00398-23-s0001.pdf, PDF file, 0.5 MB [file spectrum.00398-23-s0001.pdf]

SUPPLEMENTARY MATERIALS

Biogeographical and biodiversity patterns of marine planktonic bacteria spanning from the South China Sea across the Golf of Bengal to the northern Arabian Sea

Lijuan Ren [a, b*], Xingyu Song [b], Chuangfeng Wu [a], Gang Li [b], Xiufeng Zhang [a], Xiaomin Xia [b], Chenhui Xiang [b], Bo-Ping Han [a], Erik Jeppesen [c, d, e], and Qinglong L. Wu [f, g]

[a] Department of Ecology and Institute of Hydrobiology, Jinan University, Guangzhou, China;

[b] Key Laboratory of Tropical Marine Bio-resources and Ecology and Key Laboratory of Science and Technology on Operational Oceanography, South China Sea Institute of Oceanology, Chinese Academy of Sciences, Guangzhou, China;

[c] Sino-Danish Centre for Education and Research, University of Chinese Academy of Sciences, Beijing, China;

[d] Department of Bioscience, Aarhus University, Silkeborg, Denmark;

[e] Limnology Laboratory, Department of Biological Sciences and Centre for Ecosystem Research and Implementation, Middle East Technical University, Ankara, Turkey;

[f] Center for Evolution and Conservation Biology, Southern Marine Sciences and Engineering Guangdong Laboratory (Guangzhou), Guangzhou, China;

[g] State Key Laboratory of Lake Science and Environment, Nanjing Institute of Geography and Limnology, Chinese Academy of Sciences, Nanjing, China.

Corresponding authors: Lijuan Ren, Email: lijuanren@jnu.edu.cn

Running title: Microbial diversity and chlorophyll *a* relationships

Table S1 Spearman's rho rank correlation coefficients between pairwise environmental factors. T: temperature, SiO_3^{2-}: silicate, PO_4^{3-}: phosphate, TN: total nitrogen, NO_3^-: nitrate, NO_2^-: nitrite, NH_4^+: ammonium, chlorophyll *a*: the measured chlorophyll *a* concentration, annual chlorophyll *a*: the annual chlorophyll *a* before the sampling time, annual T: the annual temperature before the sampling time, annual POC: the annual particulate organic carbon before the sampling time. *: $p < 0.05$; **: $p < 0.01$.

Index	T	Salinity	SiO_3^{2-}	PO_4^{3-}	TN	NO_2^-	NO_3^-	NH_4^+	Chlorophyll *a*	Annual chlorophyll *a*	Annual T
T											
Salinity	-0.47**										
SiO_3^{2-}	-0.36*	0.12									
PO_4^{3-}	-0.77**	0.77**	0.486**								
TN	-0.71**	0.63**	0.611**	0.9**							
NO_2^-	-0.41**	0.76**	0.463**	0.72**	0.72**						
NO_3^-	-0.69**	0.65**	0.594**	0.91**	0.997**	0.72**					
NH_4^+	0.14	-0.32*	0.022	-0.29	-0.07	-0.27	-0.14				
Chlorophyll *a*	-0.77**	0.63**	0.561**	0.90**	0.82**	0.68**	0.83**	-0.335*			
Annual chlorophyll *a*	-0.73**	0.64**	0.57**	0.93**	0.89**	0.68**	0.9**	-0.152	0.92**		
Annual T	0.89**	-0.52**	-0.198	-0.79**	-0.75**	-0.38*	-0.75**	0.199	-0.76**	-0.76**	
Annual POC	-0.75**	0.67**	0.61**	0.95**	0.93**	0.73**	0.93**	-0.202	0.94**	0.98**	-0.76**

Table S2 Relating the investigated environmental variables and geographical factors to the regional distributions of both particle-associated and free-living bacterial community compositions based on multiple regression on distance matrices (MRM). Permutation tests of significance for regression coefficients and R-squared were used in MRM. PACCs: particle-associated bacterial community compositions and FLCCs: free-living bacterial community compositions.

Index	PACCs		FLCCs	
	R^2	*p*	R^2	*p*
Pure environmental variables (penv)	0.041	0.01**	0.206	0.01**
Pure geographical distances (pgeo)	0.231	0.01**	0.156	0.01**
Mix of penv and pgeo	0.120	0.01**	0.199	0.01**
Residual	0.608		0.439	

**: $p < 0.01$; *: $p < 0.05$

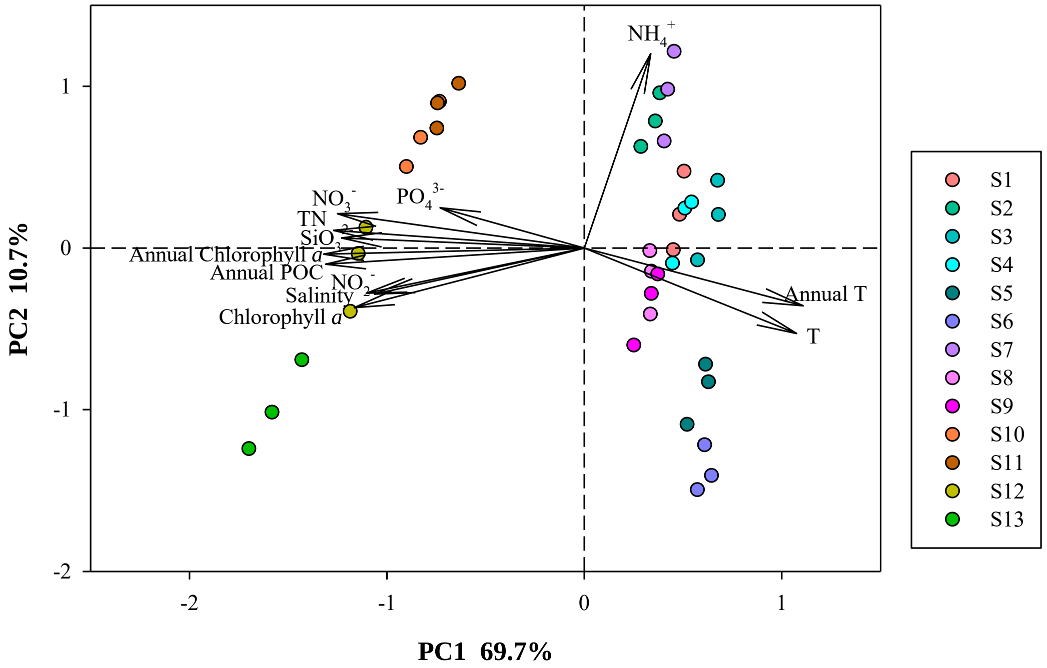

Fig. S1 Principal component analysis (PCA) depicting the relationships among all of the investigated environmental variables. T: temperature, SiO_3^{2-}: silicate, PO_4^{3-}: phosphate, TN: total nitrogen, NO_3^-: nitrate, NO_2^-: nitrite, NH_4^+: ammonium, chlorophyll *a*: the measured chlorophyll *a* concentration, annual chlorophyll *a*: the annual chlorophyll *a* before the sampling time, annual T: the annual temperature before the sampling time, annual POC: the annual particulate organic carbon before the sampling time.

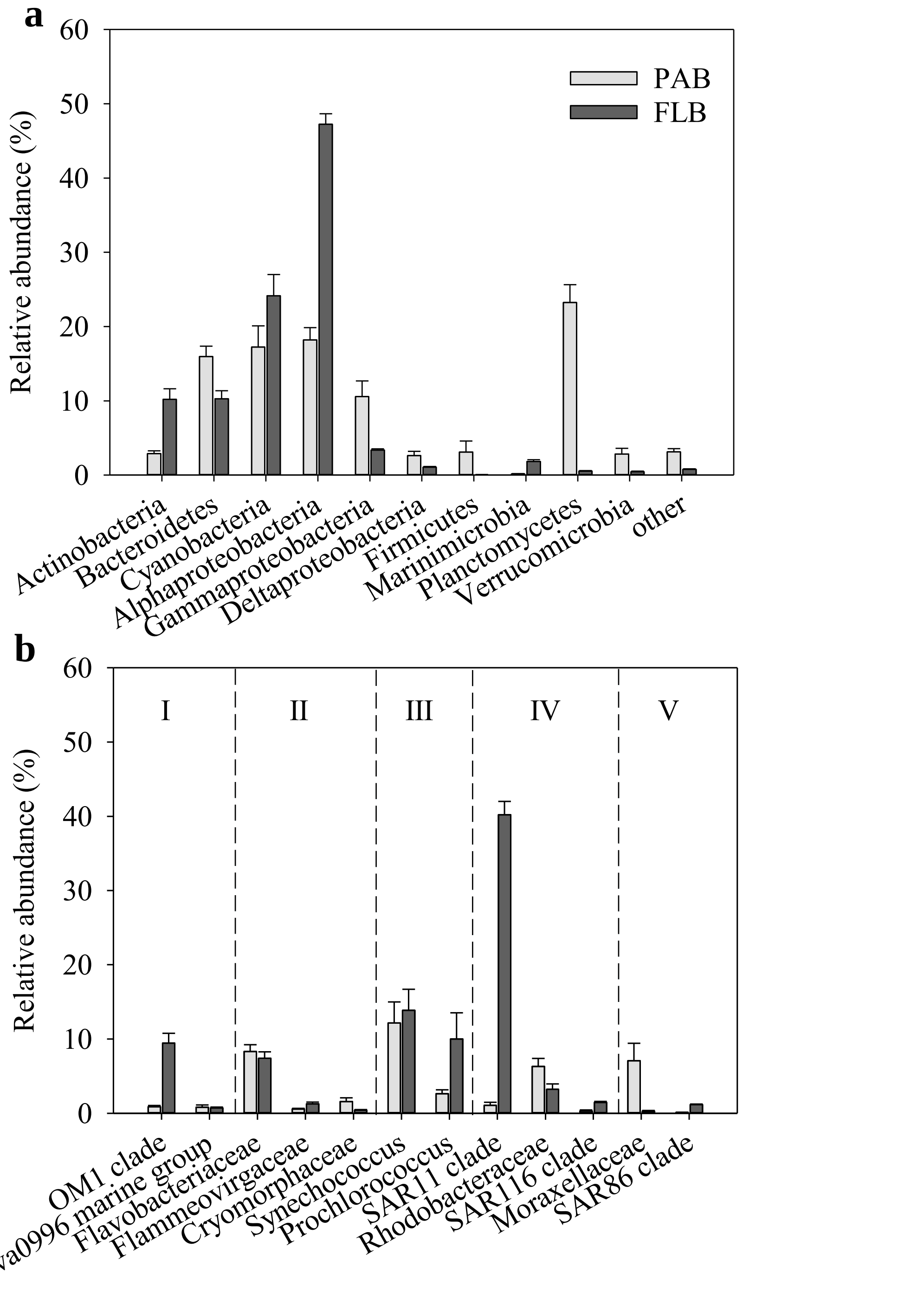

Fig. S2 The dominant (sub)phyla (a) and clades or families (b) in both particle-associated bacteria (PAB) and free-living bacteria (FLB). I: *Actinobacteria*, II: *Bacteroidetes*, III: *Cyanobacteria*, IV: *Alphaproteobacteria*, and V: *Gammaproteobacteria*.

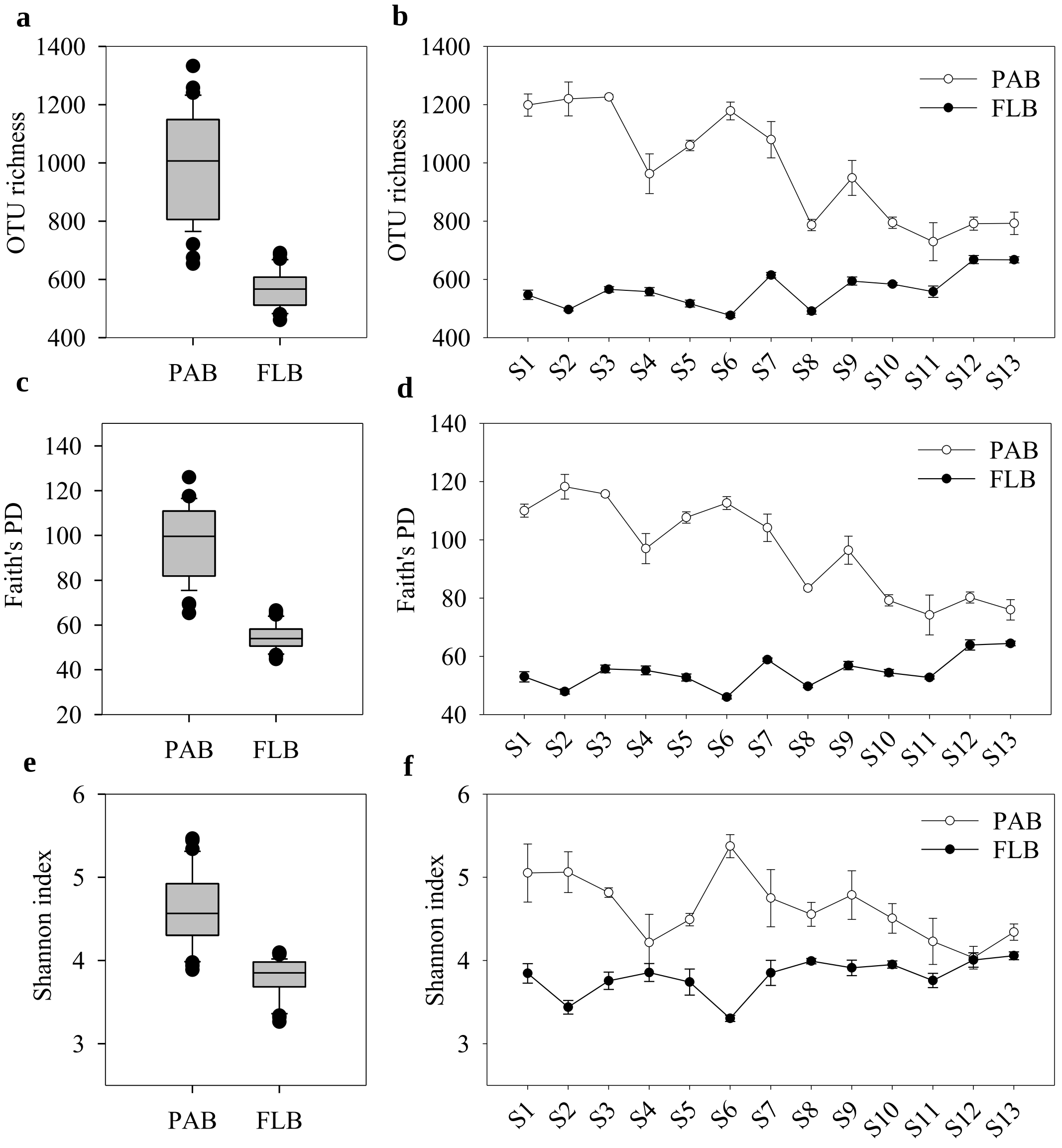

Fig. S3 The alpha diversity of OTU richness (a and b), Faith's phylogenetic diversity (Faith's PD, c and d), and Shannon index (e and f) of both particle-associated bacteria (PAB) and free-living bacteria (FLB) (a, c, and e) and their distributions across different sampling sites (b, d and f).

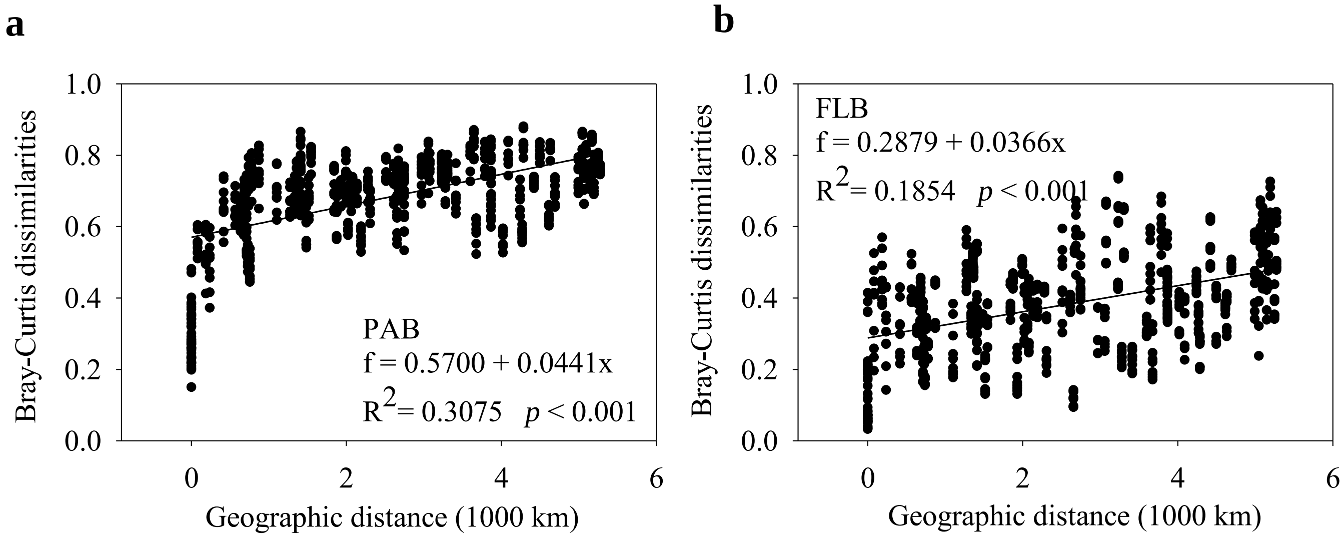

Fig. S4 The relationships between the Bray-Curtis dissimilarities of both PAB (a) and FLB (b) communities and geographic distance.

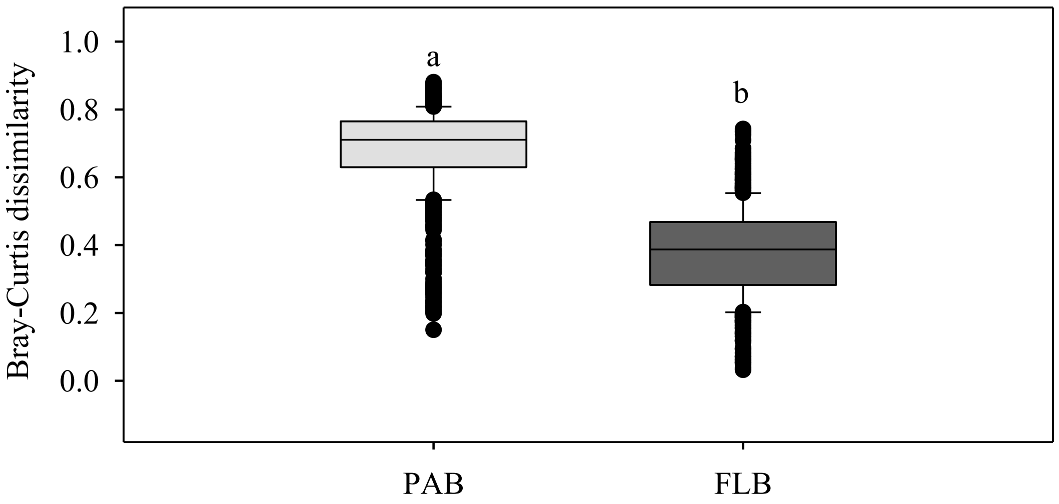

Fig. S5 The beta diversity base on the Bray-Curtis dissimilarity for both particle-associated bacteria (PAB) and free-living bacteria (FLB). Significant ($p < 0.05$) differences among groups are indicated by different alphabetic letters above the bars.

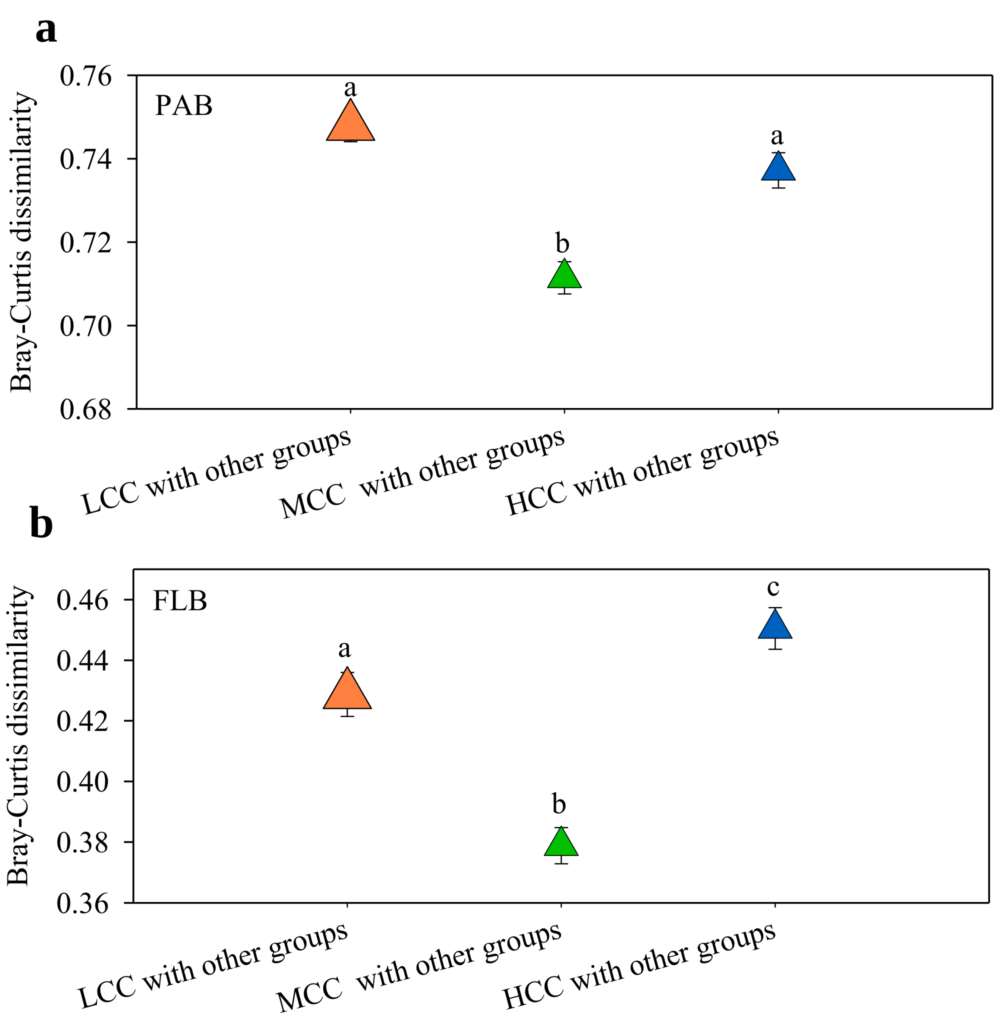

Fig. S6 Beta diversity of both PAB (**a**) and FLB (**b**) between groups of different chlorophyll *a* concentrations. LCC: low chlorophyll *a* concentrations (chlorophyll *a* < 0.2 μg L^{-1}), MCC: medium chlorophyll *a* concentrations (0.2 μg L^{-1} < chlorophyll *a* 0.5 μg L^{-1}), and HCC: high chlorophyll *a* concentrations (chlorophyll *a* > 0.5 μg L^{-1}). Significant ($p < 0.05$) differences among groups are indicated by different alphabetic letters above the bars.

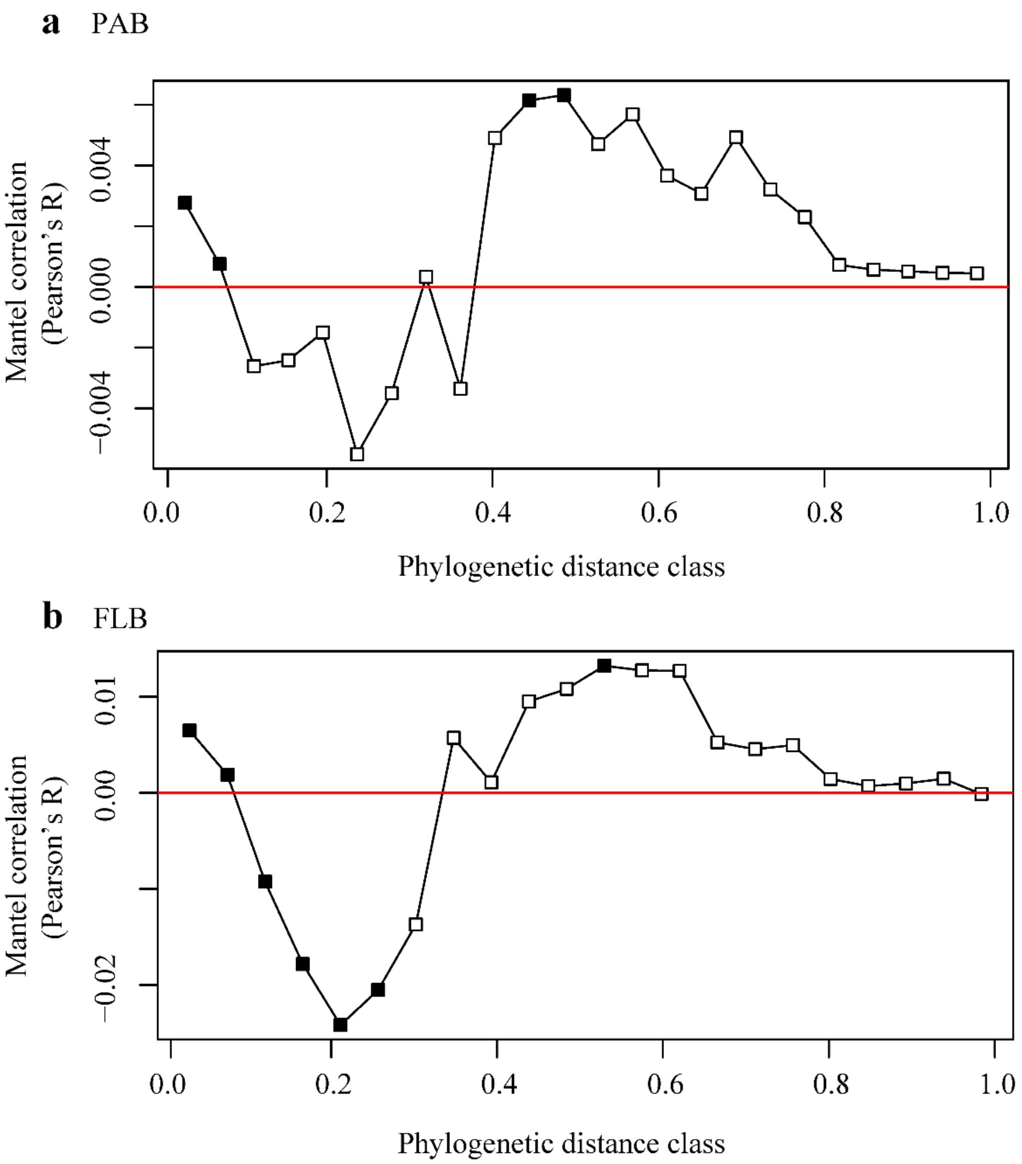

Fig. S7 Pearson's correlations from the Mantel correlogram (999 permutations) between the pairwise matrix of OTU niche distances and phylogenetic distances of both particle-associated bacteria (PAB, a) and free-living bacteria (FLB, b). The phylogenetic signal in niche associations was indicated by significant correlations ($p < 0.05$, solid circles).

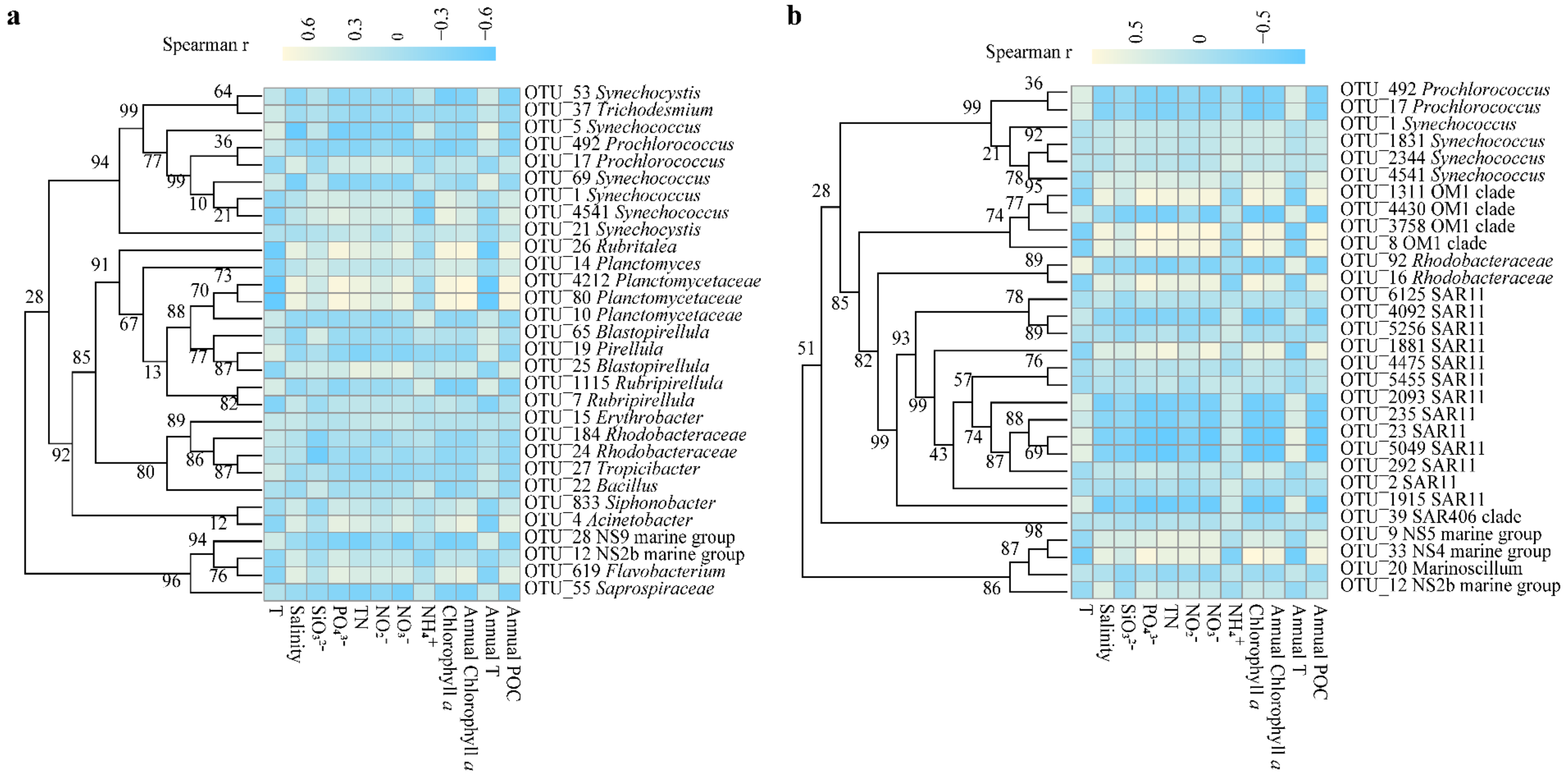

Fig. S8 The phylogenetic distribution of the top 30 most abundant taxa and their environmental preferences in both particle-associated bacteria (PAB, a) and free-living bacteria (FLB, b). The phylogenetic tree was constructed using the neighbor-joining method. Taxa that could be assigned to genus level are shown as genus, otherwise as family.

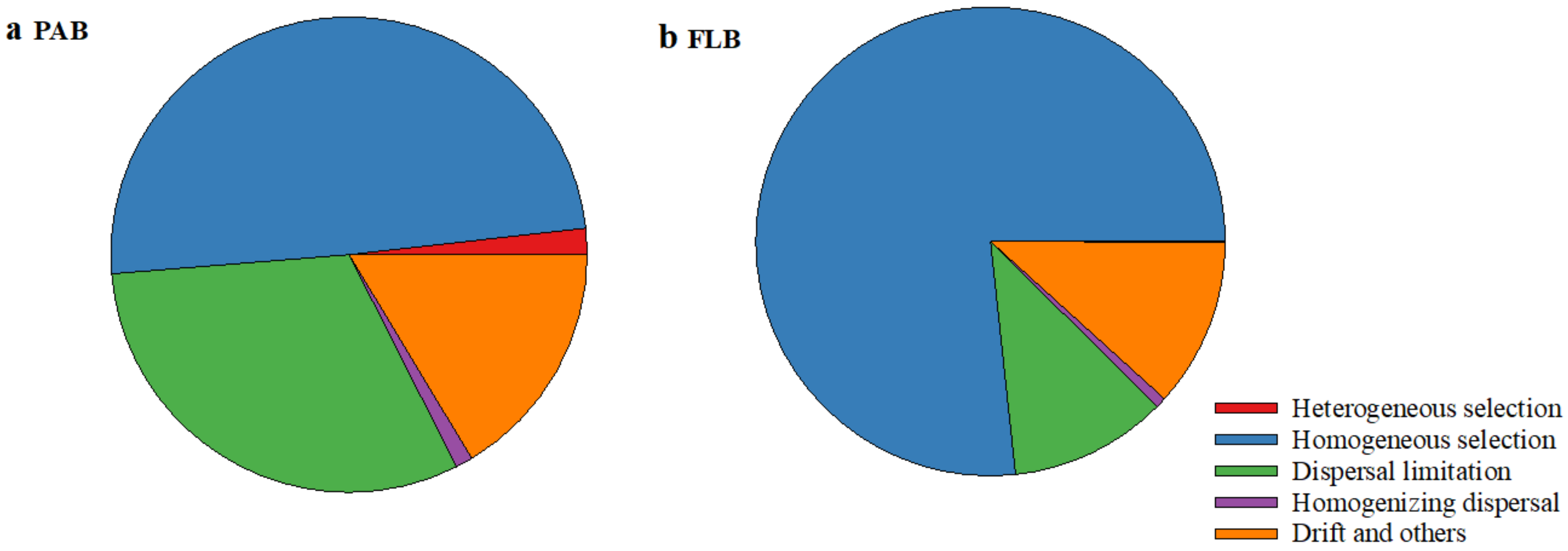

Fig. S9 The relative importance of community assembly processes of particle-associated bacteria (PAB, a) and free-living bacteria (FLB, b), respectively. The community assembly processes included homogeneous and heterogeneous selection, dispersal limitations, homogenizing dispersal and the drift and other fractions.

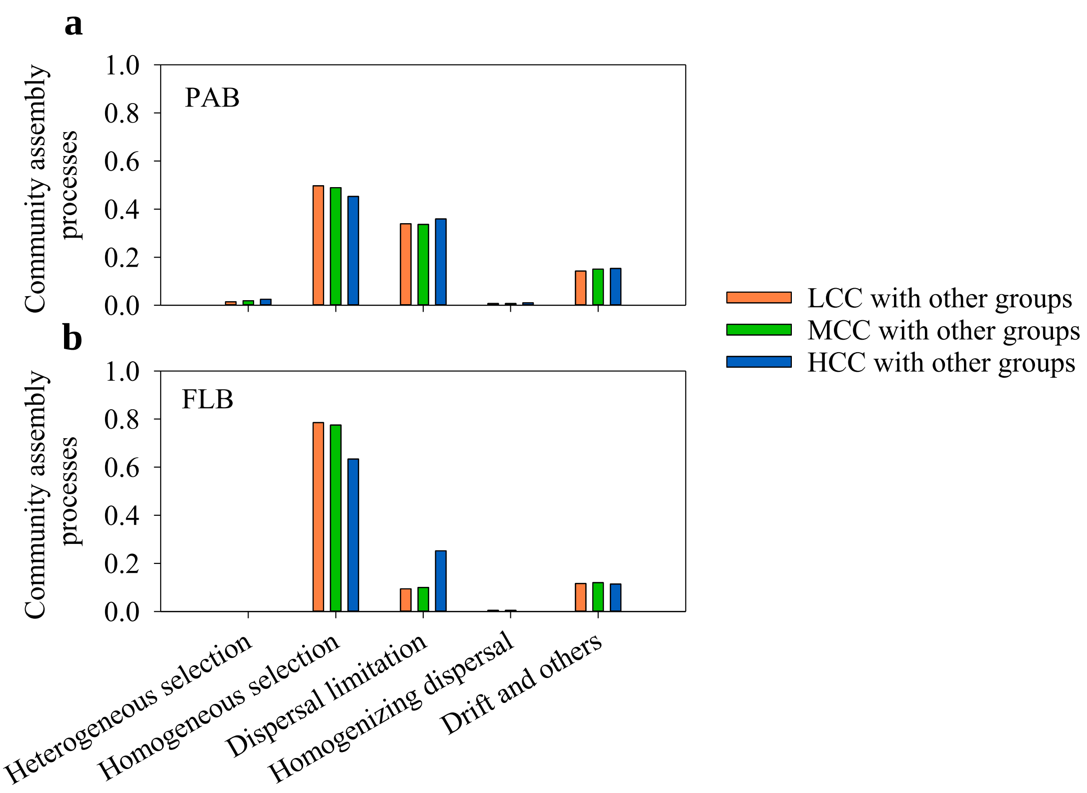

Fig. S10 The relative importance of community assembly processes of particle-associated bacteria (PAB, **a**) and free-living bacteria (FLB, **b**) between groups of different chlorophyll *a* concentrations. The community assembly processes included homogeneous and heterogeneous selection, dispersal limitations, homogenizing dispersal and the drift and other fractions. LCC: low chlorophyll *a* concentrations (chlorophyll $a < 0.2$ μg L^{-1}), MCC: medium chlorophyll *a* concentrations (0.2 μg L^{-1} chlorophyll $a < 0.5$ μg L^{-1}), and HCC: high chlorophyll *a* concentrations (chlorophyll $a > 0.5$ μg L^{-1}).
